# Highly multiplexed targeted sequencing strategy for infectious disease surveillance

**DOI:** 10.1186/s12896-023-00804-7

**Published:** 2023-08-23

**Authors:** Iván Hernández-Neuta, Anastasia Magoulopoulou, Flor Pineiro, Jan Gorm Lisby, Mats Gulberg, Mats Nilsson

**Affiliations:** 1 Department of Biochemistry and Biophysics, Faculty of Science, Stockholm University, Svante Arrhenius väg 16C, Stockholm, 104 05 Sweden; 2 Science for Life Laboratory (SciLifeLab), Tomtebodavägen 23, 171 65, Solna, Sweden; 3 Department of Clinical Microbiology, Amager and Hvidovre Hospital, University of Copenhagen, Kettegaard Alle 30, Hvidovre, 2650 Denmark; 4 Q-linea AB, Dag Hammarskjölds Väg 52A, Uppsala, 752 37 Sweden

**Keywords:** Molecular inversion probes (MIPs), Next generation sequencing (NGS), Infectious diseases, Diagnostics, Disease surveillance, Pathogen detection

## Abstract

**Background:**

Global efforts to characterize diseases of poverty are hampered by lack of affordable and comprehensive detection platforms, resulting in suboptimal allocation of health care resources and inefficient disease control. Next generation sequencing (NGS) can provide accurate data and high throughput. However, shotgun and metagenome-based NGS approaches are limited by low concentrations of microbial DNA in clinical samples, requirements for tailored sample and library preparations plus extensive bioinformatics analysis. Here, we adapted molecular inversion probes (MIPs) as a cost-effective target enrichment approach to characterize microbial infections from blood samples using short-read sequencing. We designed a probe panel targeting 2 bacterial genera, 21 bacterial and 6 fungi species and 7 antimicrobial resistance markers (AMRs).

**Results:**

Our approach proved to be highly specific to detect down to 1 in a 1000 pathogen DNA targets contained in host DNA. Additionally, we were able to accurately survey pathogens and AMRs in 20 out of 24 samples previously profiled with routine blood culture for sepsis.

**Conclusions:**

Overall, our targeted assay identifies microbial pathogens and AMRs with high specificity at high throughput, without the need for extensive sample preparation or bioinformatics analysis, simplifying its application for characterization and surveillance of infectious diseases in medium- to low- resource settings.

**Supplementary Information:**

The online version contains supplementary material available at 10.1186/s12896-023-00804-7.

## Introduction

One of the aims of Sustainable Development Goals (SDGs), set by the United Nations Assembly in 2015, is the Global under-5 mortality (U5MR) rate reduction. The desired rate to be reached by 2030 was set to a rate as low as 25 deaths in 1000 livebirths for U5MR and to as low as 12 deaths in 1000 livebirths for neonatal mortality rate (NMR)[1]. Although by 2019 U5MR has decreased by 59% since 1990 and the majority of world countries were following up on the set goals, in 2019 alone about 5,2 million children under 5 died worldwide due to preventable or treatable causes [1]. One of the leading causes of death are microbial infections, alongside with asphyxia and preterm birth complications. Additionally, the death incidents show great geographic and economic variation with Sub-Saharan Africa holding the highest rates of mortality in the world [2].

The limited infrastructure and resources available in low and middle income countries hinders the generation of accurate epidemiological data regarding the incidence and prevalence of infectious diseases. Most of the available estimates are based on verbal autopsies or extrapolated from few characterized cases rather than the result of accurate diagnostic tests or proper medical assessment [3]. One of the main limitations to generate reliable data are the high costs and limited throughput associated to methods routinely used for epidemiology and surveillance [4, 5]. Therefore, generation of representative surveillance data and thus proper resource allocation and death incidence prevention are hindered.

With the emergence of next generation sequencing (NGS) technologies, identification and discovery of microbes from clinical samples has not only become possible but also feasible for clinical implementation. Metagenomics and shotgun NGS approaches allow for identification, typing and classification of microbial communities directly from clinical samples [6]. However, these methods present a limited sensitivity as a consequence of the low concentration of microbial pathogens in most clinical samples, which biases any target amplification towards the host DNA [7]. Moreover, bioinformatics analysis becomes cumbersome since the final number of reads that can be mapped to microbial pathogen sequences are less than 1% of the total reads [8]. In order to overcome this, a number of sample preparation protocols have been developed to either enrich the pathogen DNA or to clean up the host DNA content [5, 9]. Nevertheless, these approaches significantly increase the total turn-around-time as well as the overall cost of the assay, making it financially impractical for routine surveillance, especially for developing countries [10].

On the other hand, targeted approaches allow to focus the analysis towards a number of targets of interest [11]. PCR is the most popular method to analyze a limited amount of targets, and amplification of 16 S and internal transcribed spacer (ITS) regions allow for a general microbial detection and classification approach [12]. However, limitations can be encountered due to bias during multiplex PCR amplification [13, 14]. Other target-enrichment strategies include hybridization-based methods that isolate the regions of interest by capturing them on a solid surface before library preparation. These allow for high multiplexity and can be cost effective for a high number of samples but require high amounts of purified DNA limiting its application for microbial identification.

The use of Molecular Inversion Probes (MIPs) for target enrichment is a promising approach, since it allows for a high level of multiplexing in a cost-effective manner compared to other targeted strategies [11, 15, 16]. MIPs are linear oligonucleotides with complementary ends matching a specific target. These ends or arms form an open circle structure upon hybridization, leaving a gap in between. A polymerase extends the 3’ end towards the 5’ end of the probe and a DNA ligase joins the resulting nick. The probe arms are linked with a backbone sequence that contains primer binding sites for downstream amplification and detection [17]. MIPs have been mainly used for multiplexed single-nucleotide polymorphism (SNP) genotyping, detection of low-frequency variations, allele quantification, targeted resequencing and exome sequencing [18, 19]. The input requirements for these techniques have been reported to be around 200 ng thus allowing to adapt the use of MIPs for targeted infectious disease detection, including approaches targeting 16 S ribosomal sequences and antibiotic resistance markers [20–24].

Here, we adapted MIPs and short-read single end Illumina sequencing as an approach to characterize and identify pathogens and antimicrobial resistance genes (AMRs) on archived blood culture samples to generate surveillance data. We have designed a panel comprised of 144 probes targeting 21 bacterial species, 2 bacterial genera, 6 fungi species and 7 antimicrobial resistance markers. Moreover, we have developed and automated a bioinformatics pipeline that implements the analysis with minimal computational resources. Analytical validation of the method was performed with different mixes of extracted microbial genomic DNA, which allowed for specific and robust detection of down to 1 in a 1000 pathogen DNA targets contained in host DNA. Moreover, the assay capabilities with a set of 24 DNA extracts from positive blood cultures, were further validated. We were not only able to confirm the assignment of pathogens and AMRs that were detected through blood culturing, but we also detected *E. coli* in one of the samples that was not identified by blood culture. Overall, the versatility, specificity and robustness of the presented method can be implemented for characterization and routine surveillance for infectious diseases of microbial origin.

## Materials and methods

Tth ligase (EN13-B) and dNTPs (RP65) were acquired from Blirt S. A. (Gdansk, Poland). Phusion High-Fidelity DNA Polymerase (F530L), Phusion Hot Start II DNA Polymerase (F549L), Exonuclease I (EN0582), Exonuclease III (EN0191) and SYBRgreen (S7567) were obtained from ThermoFisher Scientific (Stockholm, Sweden). Φ29 (Phi-29) DNA polymerase (4002) was obtained by Monserate Biotechnology Group (San Diego, USA). All oligonucleotides were synthesized by Integrated DNA Technologies (Coralville, USA). MgCl_2_, KCl and Triton® X-100, NAD (MN1454) and human genomic DNA (11691112001) were obtained from Sigma-Aldrich (Darmstadt, Germany). Tris-HCl and phosphate-buffered saline (PBS) were obtained from Karolinska Institute Substrat (Stockholm, Sweden). QIAamp DNA Mini Kit (51304) was obtained from Qiagen (Hilden, Germany). NextSeq 500/550 High Output Kit v2.5 (75 Cycles) (20024906) sequencing kits and PhiX Control v3 (15017666) were obtained from Illumina, Inc. (San Diego, USA).

### Isolation of microbial DNA from positive blood cultures

The isolation of microbial DNA from positive blood cultures of 5 ml volume was performed using Molzym Microbial DNA MolYsis™ Complete5 © D-321-100 (Bremen, Germany) according to manufacturer instructions. The procedure included initially the human DNA removal, then the universal lysis of Gram-negative, Gram-positive bacteria and fungi and the isolation of the microbial DNA.

### Matrix interference assessment on spiked whole blood samples

Blood samples of 200 µL volume were spiked with 100 ng of isolated DNA of *E. coli* (ATCC 25922). QIAamp DNA Mini Kit (Qiagen) was used for the isolation of microbial DNA from human blood samples according to manufacturer’s instructions.

### Target selection and probe design

**Fig. 1:**
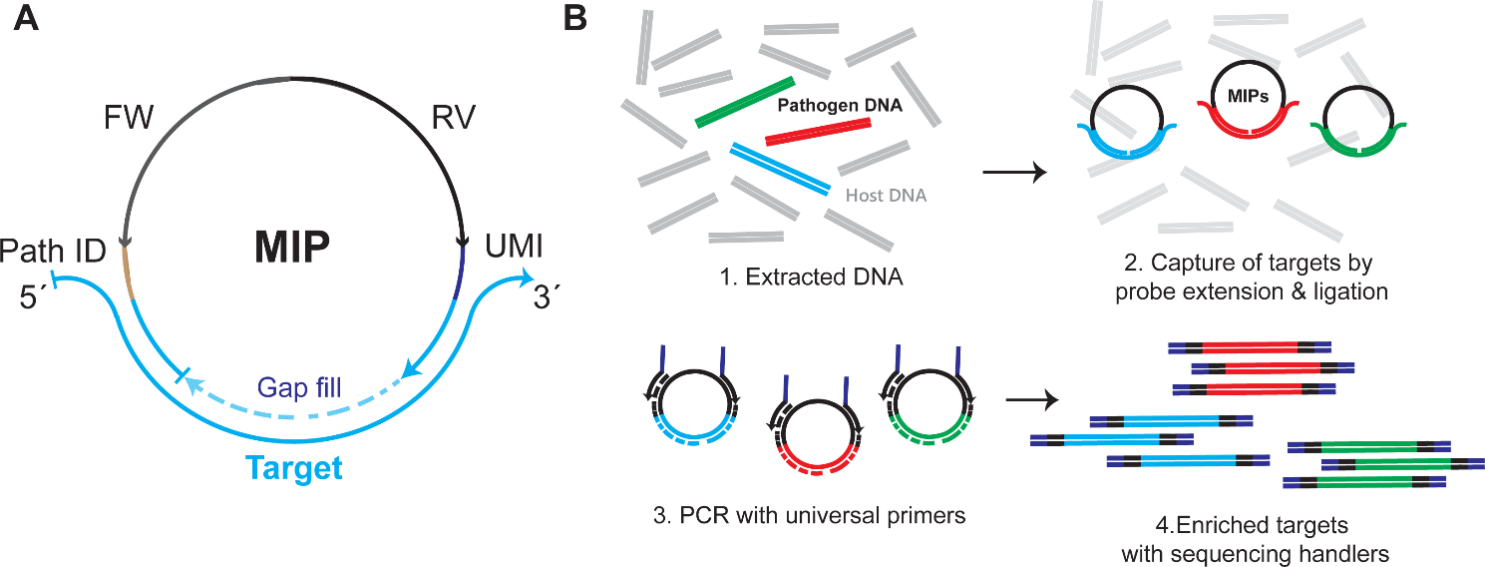
General outline of the method. **(A)** MIP arms (blue) hybridize to the complementary target regions forming an open circle. A polymerase extends the gap in between arms (dotted line) and a DNA ligase is used to close the resulting nick. The MIP backbone contains: FW & RV: Forward & reverse primer binding sites. UMI: Unique molecular identifier. Path ID: Pathogen identifier. **(B)** (1) Extracted DNA contains few copies of pathogen DNA. (2) Targets are captured by adding MIPs that circularize after extension and ligation templated by the targets. (3) & 4. After exonuclease treatment, reacted probes are amplified with universal primers containing sequencing handlers for downstream library indexing and pooling for short-read single end Illumina sequencing for pathogen identification

Bacterial Genome-sequences were downloaded from NCBI public database (Blastn/nucleotide collection: https://www.ncbi.nlm.nih.gov). The selection of species and AMR genes was based on literature on the most frequently detected microorganisms and AMRs on sepsis patient samples at the time of design (2016–2017). All the available annotated sequences for the selected species and AMRs were used for identifying 40–57 nt long preserved regions by doing pairwise alignment avoiding highly repetitive regions and homomers of more than 5 nucleotides. Identified unique targets were split in 3 parts: a 5’ arm binding region with a Tm range of 54-56^o^C; a gap-fill region of 10 nt and a 3’ arm binding region with a Tm range of 79-84^o^C. Both probe arms were connected with a backbone containing a 5 nt pathogen ID, a 15 nt forward primer sequence, a 15 nt reverse primer sequence and a 9 nt UMI (as indicated in Fig. 1A). No model organism was used to select the specific targets and probe sequences since such approach would risk not finding actual clinical findings due to the variability in genome sequence among bacteria belonging to the same species. Full list of target sequences and designed molecular inversion probes for the identification of microbes and antibiotic resistance genes are provided in Suppl. Tables 1 and 2, respectively.

### Target enrichment

The MIP-based target enrichment protocol described below is based on previously reported protocols for genomic DNA and cDNA [15, 22]. The gap-fill ligation mix contained a final concentration of 100 pM each MIP (14.5 nM total), 125 nM dNTPs, 250 mU/µL Tth ligase, 20 mU/µL Phusion High-Fidelity DNA Polymerase, 1x Ampligase buffer (20 mM Tris-HCl (pH 8.3), 25 mM KCl, 10 mM MgCl_2_, 0.5 mM NAD, 0.01% Triton® X-100) and 5 µL of DNA extract in a final volume of 20 µL. The mix was incubated for 3 min at 98 °C followed by 45 min at 55 °C, 20 min at 72 °C and hold at 12 °C or cooled on ice before the exonuclease treatment. Exonuclease treatment was performed by adding exonuclease I and exonuclease III to a concentration of 0.3 U/µL and 1.8 U/µL respectively, in a final volume of 25 µL followed by an incubation at 37 °C for 45 min and inactivation at 80 °C for 20 min. 10 µL of each exonuclease treated sample was processed immediately after by real-time PCR using final concentrations of 500 nM of Forward and Reverse primers, 0.5x SYBR green, 200 µM dNTPs, 20 mU/µL Phusion Hot Start II DNA Polymerase and 1x HF buffer in a final volume of 20 µL. The enrichment PCR was programmed as follows: (1) 98 °C for 2 min, (2) 98 °C for 10 s, (3) 55 °C for 30 s, (4) 72 °C for 30 s, (5) return to step 2 for a total of 30 cycles, (6) 72 °C for 2 min. Amplicons were stored at − 20 °C until use.

### Sample indexing and library preparation

1 µL of the enriched amplicons were indexed using 200 µM TrueSeq HT combinatorial dual (CD) index adapters (Supp. Table 3), 200 µM dNTPs, 20 mU/µL Phusion Hot Start II DNA Polymerase and 1x HF buffer to a final volume of 10 µL with a PCR program that includes: (1) 98 °C for 2 min, 2 cycles at (2) 98 °C for 15 s, (3) 60 °C for 1 min, (4) 72 °C for 1 min, (5) and final extension at 72 °C for 3 min. Indexed samples were finally amplified using 100 µM P5 and P7 primers, 200 µM dNTPs, 0.5x SYBR green, 20 mU/µL Phusion Hot Start II DNA Polymerase and 1x HF buffer in a final volume of 10 µL with a real-time PCR program that includes: (1) 98 °C for 2 min, 20 cycles of (2) 98 °C for 15 s, (3) 60 °C for 1 min, (4) 72 °C for 1 min, (5) and final extension at 72 °C for 2 min. 5 µL of the resulting PCR products were pooled together and 200 µL of the resulting library was purified by adding 360 µL of AMPureXP beads (A63881) (Beckman Coulter, USA) and incubating at room temperature for 10 min. The tube was placed on a magnetic rack during 5 min to collect the beads and the supernatant was discarded. Collected beads were washed twice with 700 µL of 70% Ethanol and let dry at room temperature for 3 min. DNA was eluted by adding 50 µL mQ H_2_O and quickly vortexing and incubating for 3 min. Beads were collected by spinning down and placing the tube in the magnetic rack for 1 min. The supernatant was transferred to a new tube. The average size and integrity of the library (250 bp) was confirmed by gel electrophoresis in a 1.5% agarose gel and the concentration was measured twice by Qubit 2 using the dsDNA HS ASSAY kit (Life technologies, USA, Q32854). The library was normalized to 4 nM in RSB buffer and denatured following recommended protocols by Illumina. 10–30% of 4 nM PhiX control was spiked into the library before the run. Libraries were sequenced using Illumina Nextseq500 sequencing platform and performed with 1 × 76 cycles, and an 8 bp dual index read.

### MIP analysis

A Perl (version 5.26.3) based pipeline using previously described modules [25] was built. The steps comprised in the pipeline are: (1) Quality filter, (2) Removing of primer sequences, (3) Size filter, (4) Find unique sequences and UMI counting, (5) Trimming UMIs and alignment to the library of targets and, (6) Group using the pathogen barcode and sum the counts for UMIs of individual probes for each pathogen. We used the approach by Arts. et al. [15] to filter possible sequencing errors in the UMIs. Briefly, UMIs were sorted by descending value of reads/UMI and only the UMIs for which the coverage value was equal or above 95% of the total reads obtained for a single MIP were counted. The pipeline is written to process demultiplexed *.FASTQ files directly providing a heat map as an output including the total number of UMIs/pathogen for each sample. The pipeline is customizable to any panel design and is available at https://github.com/Moldia/Sepsis Github repository (July 14, 2023).

## Results

### Overview of the method

The adapted MIP assay for pathogen identification is briefly illustrated in Fig. 1. MIPs targeting highly conserved regions of approximately 30 bp within the pathogen genomes and AMRs were selected. Probes are designed to form a gap of 10 bp between hybridized arms and their backbone to contain: (i) forward and reverse primer sites, (ii) a 5 nt pathogen barcode, common to all probes targeting the same microbe or AMR, and (iii) a 9 nt unique molecular identifier (UMI) to eliminate PCR duplicates in the analysis (Fig. 1A). Primers are comprising a probe binding site and sequencing adaptors.

Target regions are captured by mixing the extracted DNAs from cultured strains, mocked samples or positive blood cultures with the MIP pool, Phusion polymerase and a thermostable DNA ligase. Non-reacted probes are digested by exonuclease treatment and circularized probes are amplified by qPCR. The resulting amplicons containing the sequencing adaptors are indexed, pooled, purified and normalized for single-end 75 bp Illumina sequencing (Fig. 1B).

Demultiplexed FASTQ-files are directly input into an automated pipeline that after quality filtering and primer sequence trimming, quantifies the number of UMIs/probe and align the sequences to the library of targets to finally provide the total number of UMIs/Pathogen/sample.

### Optimization and analytical validation

As reported previously, the sensitivity of an MIP-based assay is highly dependent on the probe and dNTPs concentration, and type of polymerase used in the capture step. These parameters were tested finding an optimal concentration of probes between 1 nM and 100 pM for each probe (Supp. Figure 1 A) similar to previously reported concentrations for padlock probes and other MIP-based assays [20, 22, 23]. Phusion polymerase and Klenow fragment polymerase have been reported for MIP-based assays to be able to perform the gap-fill reaction efficiently. Phusion and Klenow fragment polymerases were compared and similar to a previous report[26], we confirmed that Phusion polymerase is the most efficient to perform the gap-fill reaction (Supp. Figure 1B). On the other hand, dNTPs concentration was observed to have a minimal effect in the performance within the range tested in this study (Supp. Figure 1 C).

**Fig. 2:**
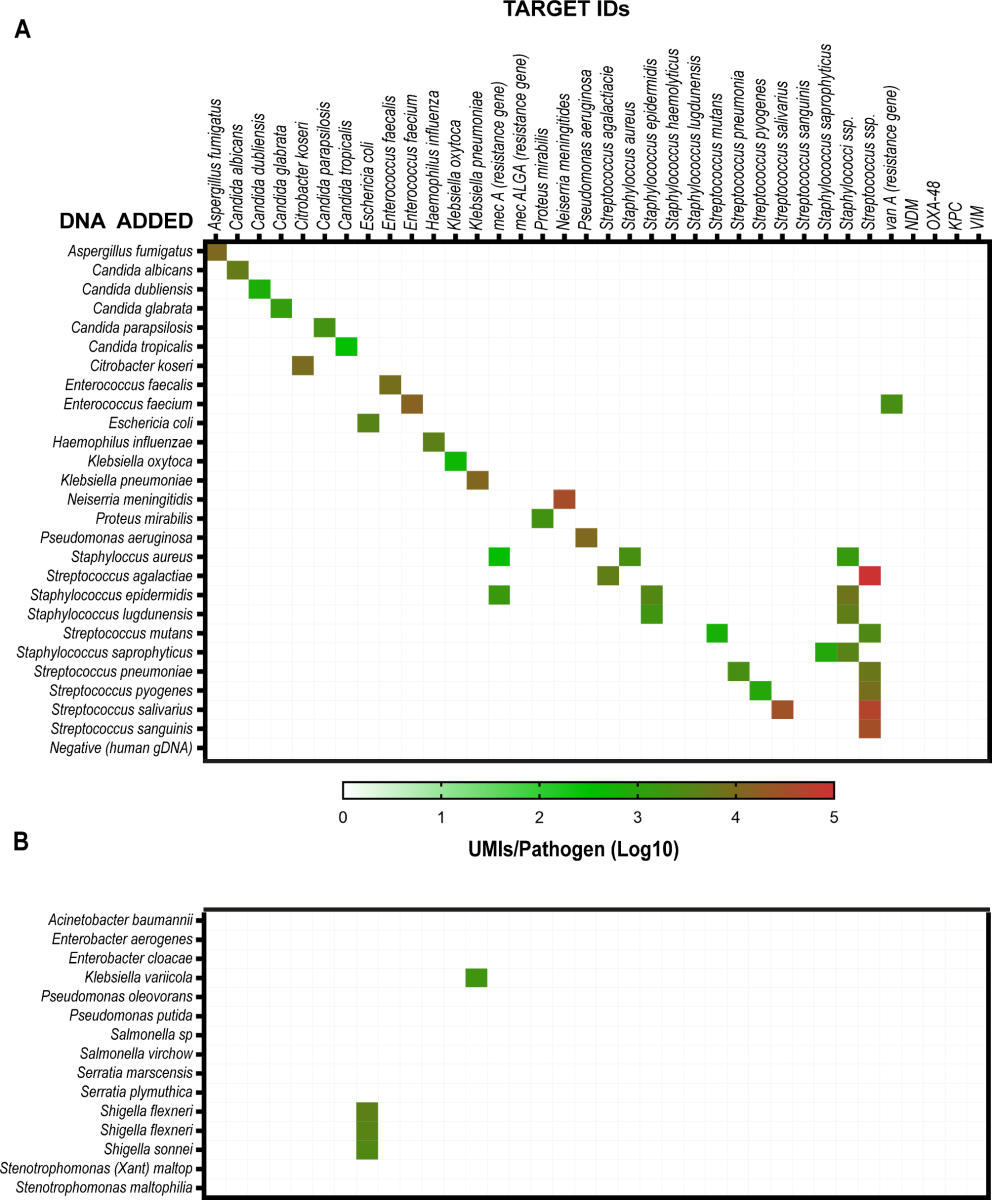
Analytical specificity of the designed MIP panel. **(A)** DNA extracts (0.1–10 ng) from individual pathogens included in the panel were tested with the assay to assess specificity and cross-reactivity (n = 4). **(B)** DNA extracts (0.1–10 ng) from species not included in the design were additionally tested to challenge the specificity of the method (n = 3)

With these optimized conditions, the specificity of the MIP panel was examined. Extracted DNA from most of the pathogen species included in the panel was obtained and each specific DNA was individually tested using the complete probe panel and adding human genomic DNA to assess its interference. The assay successfully detects individual pathogen species DNA with no cross-reactivity and no signal in those that just contained human genomic DNA (Fig. 2A). We further challenged the specificity of the probes, using DNA from pathogen species not included in the design but phylogenetically related to those included in the panel and associated to other pathologies (i.e. ventilator associated pneumonia). While the high specificity of most of the probes was confirmed, those designed for *E. coli* and *K. pneumonia* also detected closely related species such as *Shigella ssp.*. and *K. variicola* respectively (Fig. 2B). This was further confirmed *in silico* where the target sequences of these probes were also found with 100% match in the genomes of the latter species.

**Fig. 3:**
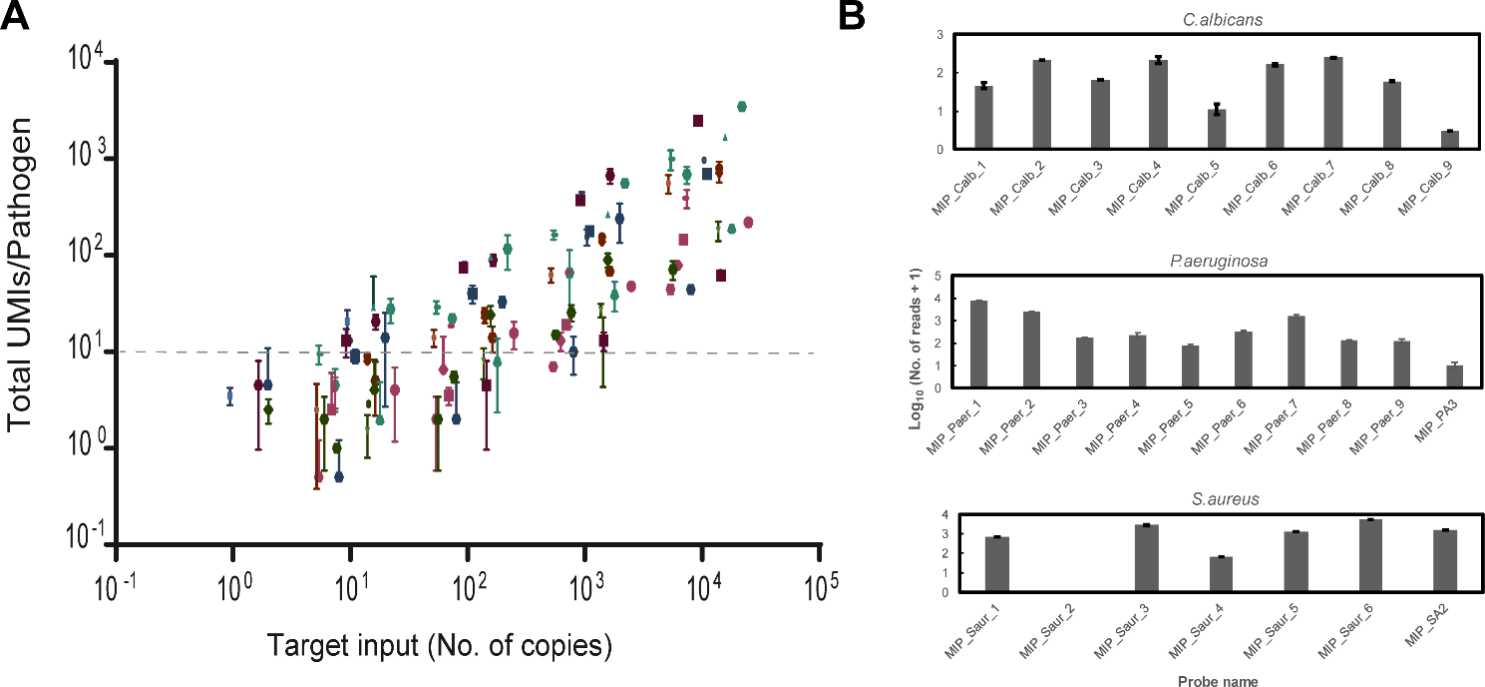
Analytical sensitivity of the method. **(A)** Serial dilutions (1:10) of mixes containing 1–10.000 genome copies of different pathogens (0.04–100 pg) were tested with the assay. 450 ng (10^4^ copies) of human genomic DNA were spiked to all mixes. Each colored symbol represents one pathogen or AMR included in the panel. Error bars correspond to standard deviation (n = 4). Dashed line indicates the set sensitivity limit of 10 UMIs/pathogen. **(B)** Examples of UMI counts for individual probes for the selected pathogens *Candida albicans*, *Pseudomonas aeruginosa* and *Staphylococcus aureus* (n = 3)

The sensitivity of the probes was further assessed by preparing serial dilutions of mixes of DNA containing 1–10.000 genome copies from different pathogens (0.04–100 pg). In addition, 450 ng (10^4^ copies) of human genomic DNA were spiked to all mixes, with the aim of assessing the capability of the method to detect and differentiate pathogens in samples that contain more than one microbe at different concentrations. Sensitivity limits ranging from 10 to 900 copies of target input were obtained (Fig. 3A), similar to previously reported levels [22]. The difference between the sensitivity levels of different pathogens can be accounted by the number of probes designed for each pathogen (Suppl. Table 4), the efficiency of individual probes (Fig. 3B) and the low coverage (1 to 2 reads/UMI) of the sequencing at low concentrations (Fig. 3A).

### Performance assessment with positive blood cultures

**Fig. 4:**
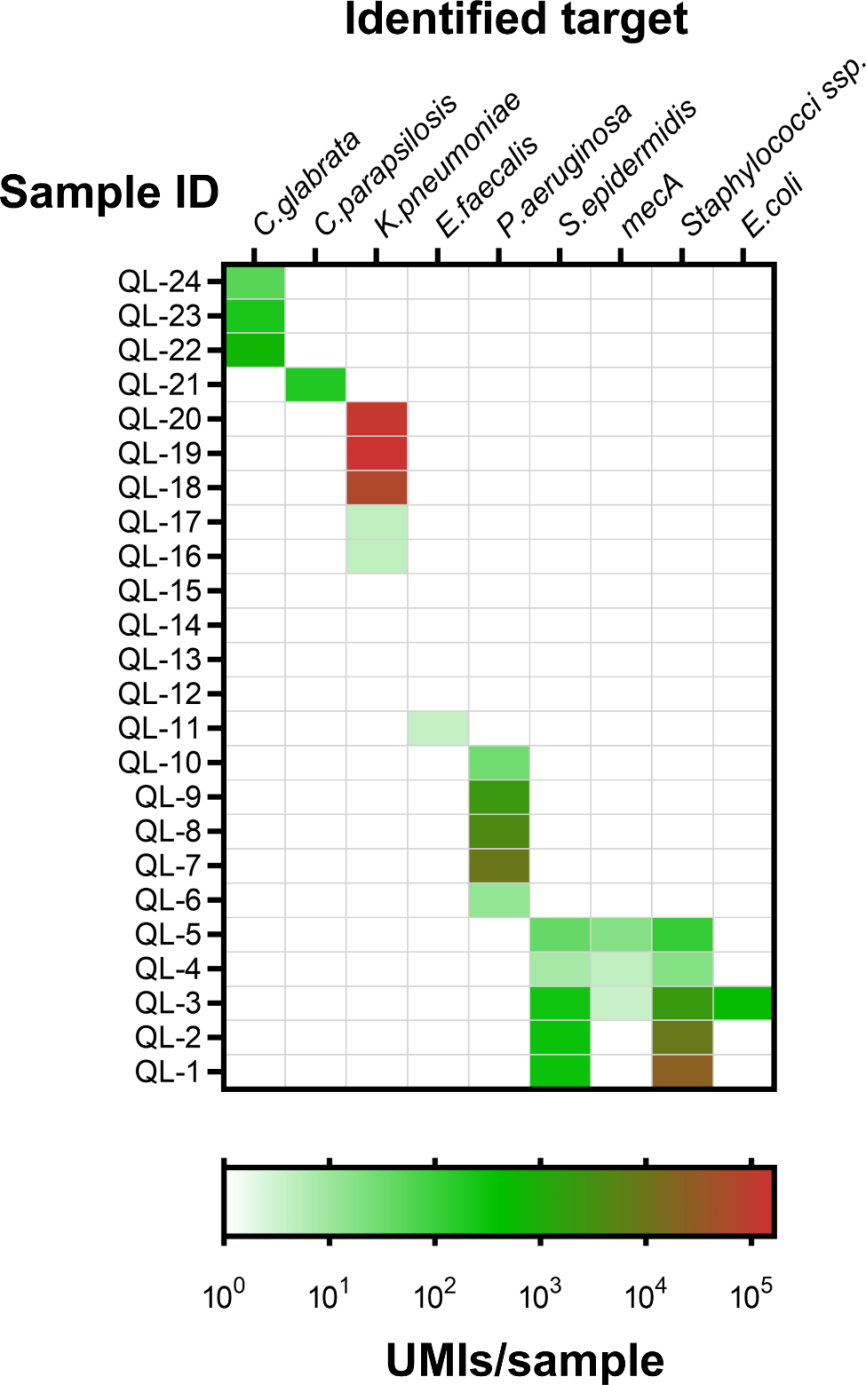
Summary results on assay performance on 24 characterized DNA extracts from positive blood cultures (n = 2). The 144 MIP panel was used and the positives results are shown (Detailed table in Supp. Table 5)

The performance of the method was further assessed by testing 24 characterized DNA extracts from positive blood culture samples containing some of the pathogens included in the panel (Suppl. Table 5). Pathogen IDs and AMRs identified with blood culture were detected in 20 out of 24 samples (Fig. 4). It was not possible to score four samples that were culture positive for *E. faecalis*, probably caused by DNA concentrations below the detection limit of the method. Moreover, the here presented method detected the presence of *E. coli* DNA in a sample which was negative by culture.

### Performance on spiked whole blood samples

**Fig. 5:**
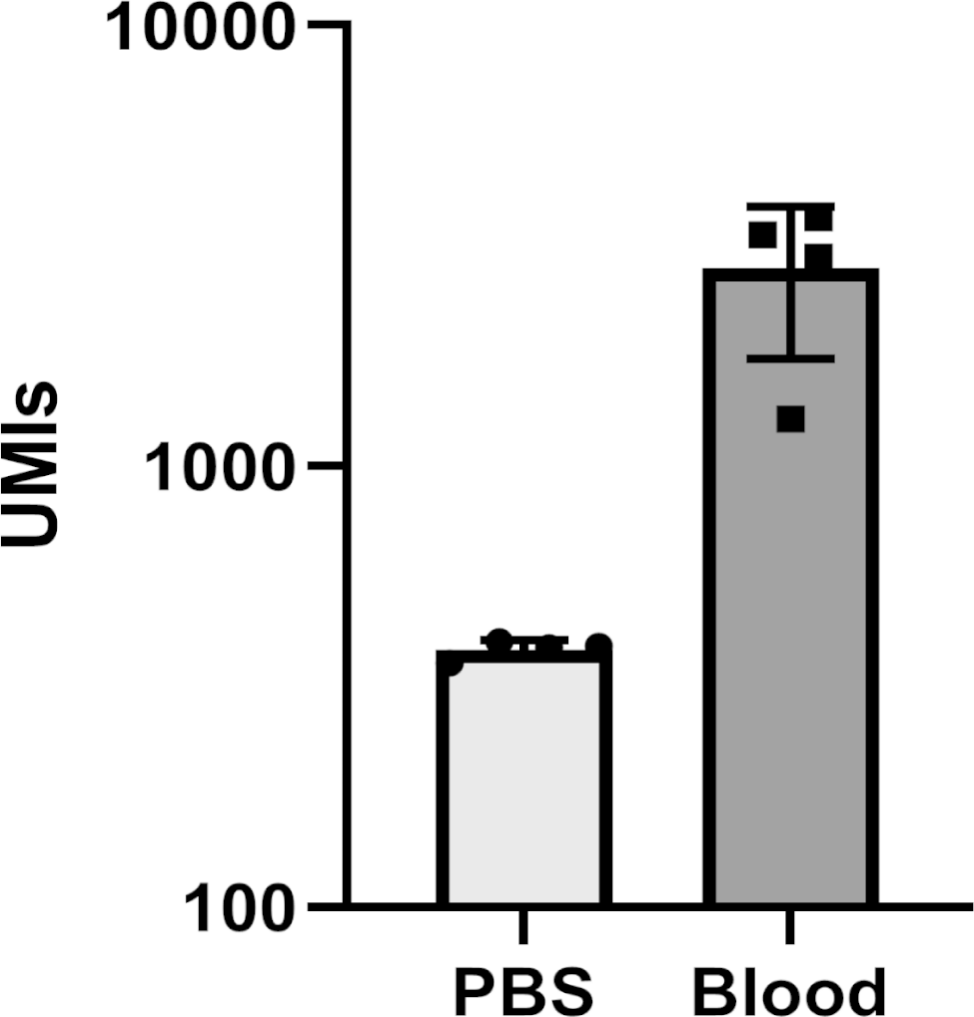
Matrix interference assessment. Number of detected UMIs from PBS (control) and whole blood samples spiked with 150 CFU/µl of *E. coli* culture (ATCC 25922) show no matrix interference (n = 4). DNA from both PBS and whole blood spiked in samples was extracted and processed with the MIP panel (144 probes)

During the optimization of the method, the effect of increasing the amount of human genomic DNA during the capture reaction was examined observing no detrimental effect on the detection of specific pathogen DNA for up to 450 ng (10^4^ copies) of human DNA present (Supp. Figure 2 A **and B**). While the extraction method used for the positive blood culture samples included a step where the human DNA is removed, we additionally examined the possibility of detecting the microbes and AMRs directly on whole blood. For this, whole blood samples were spiked with a dilution of *E. coli* (ATCC 25922) culture and compared to the performance of the assay of the same dilution but in saline buffer as control. The results showed no matrix interference or decrease in the number of reads obtained suggesting that the method can detect the spiked 150 CFUs/µL of bacteria on whole blood DNA extracts (Fig. 5).

## Discussion

Every year millions of children under the age of five die globally because of preventable or treatable causes, such as lower respiratory infections, diarrheal diseases, and other infectious diseases, with the majority of those deaths occurring in low- and middle- income countries [1, 27]. Many of these deaths are undiagnosed at the time of death and/or verbal autopsies are used for diagnosis and registration of the incidences in archives [3, 27]. Generation of surveillance data is of outmost importance for proper management and allocation of resources, risk identification and assessment of effectiveness of the current approaches [3, 4].

However, there are no high throughput methods affordable for middle- and low-income countries to achieve this, thus limiting the surveillance data to verbal autopsies or very limited data sources of a few cases [28]. NGS technologies have undergone an impressive development in the last decade becoming feasible for clinical use for infectious disease detection and characterization. The possibility of generating precise data at a high throughput makes it an ideal tool for collection of surveillance data. However, when it comes to detection of microbes from blood samples, the presence of human genomic DNA and matrix interferences can affect the overall performance of NGS methods. Targeted approaches such as PCR are alternatives to overcome this limitation by focusing the sequencing analysis towards the microbial genetic material [5, 15]. One approach that has been reported to be affordable – approx. 19 US/sample [15]- at a high throughput and high multiplex capability is the use of MIPs [15]. In this study, we adapted these probes in combination with short-read single-end NGS as a surveillance method for targeted microbial analysis on cultured blood samples.

To achieve this, a customized panel of probes targeting a wide range of microbes was designed including 2 bacterial genera, 21 bacterial species, 6 fungi species and 7 antimicrobial resistance markers. The probes are targeted to capture highly conserved regions within the microbial genomes in order to increase the specificity of the assay [23] and to minimize the bioinformatics resources required for the analysis. Furthermore, the developed analysis pipeline was designed to only count those reads that are 100% identical to the library of targets, which could also limit the number of sequencing errors or mutations within the gap. The specificity of the method was confirmed using genomic DNA extracts from the targeted microbes. The obtained results suggest that the specificity is mainly limited by the target selection for probe design and the number of conserved regions that can be found to differentiate species, especially those closely related (Fig. 2). To overcome this, sequence alignments for target selection can include not only clinically relevant strains but also related species of the natural microbiota and additionally increased stringency of the probe design pipeline is required in order to be able to find exclusive unique targets for each species. Furthermore, continuous updating with the growing number of sequences reported in the databases is also required.

The analytical sensitivity obtained in this study (Fig. 3**)** is in agreement with previous reports using MIP-based approaches [23] and sufficient to characterize samples with high microbial load (more than 100 copies/µL). A strategy that can be implemented for future assay designs would be to change the primer and probe design such that one of the sequencing handles is used as a forward primer, and the UMIs are moved in the probes to end up in the 5’ end of the reads (Supp. Figure 3 A). This increases the diversity of the library and consequently the number of UMIs up to 10 times (Supp. Figure 3B) providing increased robustness when detecting low target concentrations. The addition of rolling circle amplification (RCA) before the enrichment qPCR, or as an alternative to the exonucleolytic treatment step, could potentially increase the coverage and sensitivity without introducing bias (Supp. Figure 4 A **and B**).

When 24 characterized DNA extracts from positive blood cultures of sepsis patients were processed, we were not only able to confirm the presence of pathogens and AMRs in 20 out of the 24 samples, but also to identify *E. coli* DNA in one of the DNA extracts where blood culture had failed to identify this species (Fig. 4). As has been reported in literature [29] the sensitivity of NGS technologies to detect pathogens is higher compared to routine blood culture. Since the information we have on the tested blood samples is based only on blood culture results, it is not possible to exclude the possibility of a false positive result. Nevertheless, we should also take into consideration that antibiotics are commonly administered to patients prior to the development or worsening of sepsis related symptoms and prior to blood culture testing [30]. This can result in under detected microorganisms in blood culture testing that can be potentially detected with NGS due to the higher sensitivity of the method. The failed detection of *E. faecalis* in the four samples was possibly due to DNA from the bacterium being present in the DNA extracts at levels below to the assay’s detection limit equivalent to 100 DNA copies of target input.

Finally, matrix interference was not observed from the comparison of whole blood and saline buffer samples both spiked in with 150 CFUs/µL of *E. coli* broth culture. The DNA extracts from the spiked in blood samples produced 10 times more UMIs compared to the DNA extracts of the saline buffer spiked in samples. We hypothesize that this difference is likely caused by the commercial extraction kit used that was optimized for blood samples rather than purely saline solutions. These preliminary findings suggest that the method is also functional on whole blood DNA extracts and with further optimization could be also used as a direct from blood microbial characterization method (Fig. 5).

We observed that increasing the amount of human DNA in the capture reaction does not affect the performance of the assay (Supp. Figure 2). This result suggests that it may not be necessary to eliminate the human genomic DNA in the sample, thus indicating the target enrichment capabilities of the approach. However, this needs further testing with more microbial pathogens in a larger study.

## Conclusion

This work demonstrates the analytical properties and functionality of a MIP-based NGS assay for targeted microbial surveillance. The proposed assay is highly specific, cost-effective and requires minimal bioinformatics resources. Furthermore, the proposed assay offers great flexibility by creating customized panel(s) of MIP probes based on local epidemiology needs (bacterial and/or fungal species, AMRs etc.) or by creating larger panels including more targets. For example, more fungal species can be added to the panel, such as the *Fusarium* species *Fusarium solani*, *Fusarium oxysporum* and *Fusarium verticillioides*, which are the most common causes of fungemia after infection with *Aspergillus fumigatus* and *Candida* species [31, 32]. Additionally, the described MIP-based assay can be used for characterization of archival samples, in contrast with other methods (e.g. MALDI-TOF) that require sample cultures and input material isolation as fresh as possible [33] https://doi-org.ezp.sub.su.se/10.1007/s00253-011-3783-4.

With further validation and full automation of the library preparation, we believe that the described method can serve as a means of generating the high throughput and quality data that is required for infectious disease surveillance in developing countries. We acknowledge that NGS facilities are not always accessible to low- and middle- income countries. However, the cost for NGS has been continuously declining and due to recent outbreaks worldwide (e.g. Ebola, Zika, COVID-19) more sequencing platforms are available in local setups [33, 34]. The presented assay can identify pathogens and AMRs from positive blood cultures. Blood culture tests are performed routinely. Since our suggested MIP-based assay requires low amounts of input DNA, even leftovers could be collected (blood or DNA extracted samples) and analyzed in centralized healthcare facilities with a focus on confirming the previously given diagnosis (if any) and properly register incidents in records. Nevertheless, there are still challenges regarding shipping of the material since it would be required to keep a cold-chain. Therefore, either using DNA extracts that are less sensitive to the transport conditions or further developing the proposed method to work with dried blood spots (DBS) would significantly increase the feasibility of using this method under the proposed settings.

Among the benefits of surveillance and epidemiological data collection, prioritization of vaccine development candidates would benefit as well, providing data on incident rates, AMRs and an estimation of the number of deaths a vaccine could prevent.

### Electronic supplementary material

Below is the link to the electronic supplementary material.

Supplementary Material 1

Supplementary Material 2

Supplementary Material 3

Supplementary Material 4

Supplementary Material 5

Supplementary Material 6

Supplementary Material 7

Supplementary Material 8

Supplementary Material 9

## Data Availability

The sequences of pathogens and antimicrobial resistance markers were retrieved from NCBI database (Blastn/nucleotide collection: https://www.ncbi.nlm.nih.gov). Full list of target sequences and accession numbers is available in Supplementary Tables 1 and full list of molecular inversion probe sequences is available in Supplementary Table 2. The datasets generated during the current study are available from the corresponding author on reasonable request.

## Abbreviations

NGS: next generation sequencing
MIP(s): molecular inversion probe(s) (MIPs)
AMRs: antimicrobial resistance markers
SDGs: Sustainable Development Goals
U5MR: Global under-5 mortality
NMR: neonatal mortality rate
ITS: internal transcribed spacer
PCR: polymerase chain reaction
SNP: single nucleotide polymorphism
UMI(s): unique molecular identifier

## References

[1] Sharrow D, Hug L, You D, Alkema L, Black R, Cousens S (2022). Global, regional, and national trends in under-5 mortality between 1990 and 2019 with scenario-based projections until 2030: a systematic analysis by the UN Inter-agency Group for Child Mortality Estimation. Lancet Glob Health.

[2] Saugstad OD (2011). Reducing global neonatal mortality is possible. Neonatology.

[3] Lozano R, Freeman MK, James SL, Campbell B, Lopez AD, Flaxman AD (2011). Performance of InterVA for assigning causes of death to verbal autopsies: multisite validation study using clinical diagnostic gold standards. Popul Health Metr.

[4] Nsubuga P, White ME, Thacker SB, Anderson MA, Blount SB, Broome CV et al. Public Health Surveillance: A Tool for Targeting and Monitoring Interventions. In: Jamison DT, Breman JG, Measham AR, Alleyne G, Claeson M, Evans DB, editors. Disease Control Priorities in Developing Countries. 2nd edition. Washington (DC): World Bank; 2006.

[5] Maljkovic Berry I, Melendrez MC, Bishop-Lilly KA, Rutvisuttinunt W, Pollett S, Talundzic E (2020). Next generation sequencing and Bioinformatics Methodologies for Infectious Disease Research and Public Health: approaches, applications, and considerations for development of Laboratory Capacity. J Infect Dis.

[6] Gu W, Miller S, Chiu CY (2019). Clinical metagenomic next-generation sequencing for Pathogen Detection. Annu Rev Pathol.

[7] Goldberg B, Sichtig H, Geyer C, Ledeboer N, Weinstock GM. Making the Leap from Research Laboratory to Clinic: Challenges and Opportunities for Next-Generation sequencing in Infectious Disease Diagnostics. mBio. 2015;6. 10.1128/mBio.01888-15 PMC4669390 26646014

[8] Frey KG, Herrera-Galeano JE, Redden CL, Luu TV, Servetas SL, Mateczun AJ (2014). Comparison of three next-generation sequencing platforms for metagenomic sequencing and identification of pathogens in blood. BMC Genomics.

[9] Grubaugh ND, Ladner JT, Kraemer MUG, Dudas G, Tan AL, Gangavarapu K (2017). Genomic epidemiology reveals multiple introductions of Zika virus into the United States. Nature.

[10] Melnikov A, Galinsky K, Rogov P, Fennell T, Van Tyne D, Russ C (2011). Hybrid selection for sequencing pathogen genomes from clinical samples. Genome Biol.

[11] Mamanova L, Coffey AJ, Scott CE, Kozarewa I, Turner EH, Kumar A (2010). Target-enrichment strategies for next-generation sequencing. Nat Methods.

[12] Quince C, Lanzén A, Curtis TP, Davenport RJ, Hall N, Head IM (2009). Accurate determination of microbial diversity from 454 pyrosequencing data. Nat Methods.

[13] Pan W, Byrne-Steele M, Wang C, Lu S, Clemmons S, Zahorchak RJ (2014). DNA polymerase preference determines PCR priming efficiency. BMC Biotechnol.

[14] Carlson CS, Emerson RO, Sherwood AM, Desmarais C, Chung M-W, Parsons JM (2013). Using synthetic templates to design an unbiased multiplex PCR assay. Nat Commun.

[15] Arts P, Van Der Raadt J, Van Gestel SHC, Steehouwer M, Shendure J, Hoischen A (2017). Quantification of differential gene expression by multiplexed targeted resequencing of cDNA. Nat Commun.

[16] Almomani R, Marchi M, Sopacua M, Lindsey P, Salvi E, de Koning B (2020). Evaluation of molecular inversion probe versus TruSeq® custom methods for targeted next-generation sequencing. PLoS ONE.

[17] Hardenbol P, Banér J, Jain M, Nilsson M, Namsaraev E, Karlin-Neumann Ga (2003). Multiplexed genotyping with sequence-tagged molecular inversion probes. Nat Biotechnol.

[18] Umbarger M, Kennedy CJ, Saunders P, Breton B, Chennagiri N, Emhoff J (2013). Next-generation carrier screening. Genet Med Off J Am Coll Med Genet.

[19] Absalan F, Ronaghi M (2007). Molecular inversion probe assay. Methods in molecular biology. (Clifton N J).

[20] Koehler JW, Hall AT, Rolfe PA, Honko AN, Palacios GF, Fair JN (2014). Development and evaluation of a panel of filovirus sequence capture probes for pathogen detection by next-generation sequencing. PLoS ONE.

[21] Peker N, Rossen JWA, Deurenberg RH, Langereis PC, Raangs EGC, Kluytmans JA (2018). Evaluation of an Accelerated Workflow for Surveillance of ESBL (CTX-M)-Producing Escherichia coli using amplicon-based next-generation sequencing and automated analysis. Microorganisms.

[22] Stefan CP, Koehler JW, Minogue TD (2016). Targeted next-generation sequencing for the detection of ciprofloxacin resistance markers using molecular inversion probes. Sci Rep.

[23] Stefan CP, Hall AT, Minogue TD. Detection of 16S rRNA and KPC genes from Complex Matrix utilizing a molecular Inversion Probe Assay for Next-Generation sequencing. Sci Rep. 2018;8. 10.1038/s41598-018-19501-z PMC5794912 29391471

[24] Veenemans J, Overdevest IT, Snelders E, Willemsen I, Hendriks Y, Adesokan A (2014). Next-generation sequencing for typing and detection of resistance genes: performance of a new commercial method during an outbreak of extended-spectrum-beta-lactamase-producing Escherichia coli. J Clin Microbiol.

[25] Rosenkranz D, Han CT, Roovers EF, Zischler H, Ketting RF (2015). Piwi proteins and piRNAs in mammalian oocytes and early embryos: from sample to sequence. Genomics Data.

[26] Chen X, Sun YC, Church GM, Lee JH, Zador AM. Efficient in situ barcode sequencing using padlock probe-based BaristaSeq. Nucleic Acids Res. 2018;46. 10.1093/nar/gkx1206 PMC5829746 29190363

[27] Perin J, Mulick A, Yeung D, Villavicencio F, Lopez G, Strong KL (2022). Global, regional, and national causes of under-5 mortality in 2000–19: an updated systematic analysis with implications for the Sustainable Development Goals. Lancet Child Adolesc Health.

[28] Kawasaki T (2017). Update on pediatric sepsis: a review. J Intensive Care.

[29] Samuel L (2023). Direct-from-blood detection of pathogens: a review of Technology and Challenges. J Clin Microbiol.

[30] Duncan CF, Youngstein T, Kirrane MD, Lonsdale DO (2021). Diagnostic Challenges in Sepsis. Curr Infect Dis Rep.

[31] Garnica M, Nucci M (2013). Epidemiology of Fusariosis. Curr Fungal Infect Rep.

[32] Muhammed M, Anagnostou T, Desalermos A, Kourkoumpetis TK, Carneiro HA, Glavis-Bloom J (2013). Fusarium infection: report of 26 cases and review of 97 cases from the literature. Med (Baltim).

[33] Stockholm University. https://doi-org.ezp.sub.su.se/10.1007/s00253-011-3783-4

[34] Okeke IN, Feasey N, Parkhill J, Turner P, Limmathurotsakul D, Georgiou P (2020). Leapfrogging laboratories: the promise and pitfalls of high-tech solutions for antimicrobial resistance surveillance in low-income settings. BMJ Glob Health.

