# Supplementary material for: Highly multiplexed targeted sequencing strategy for infectious disease surveillance
Source: BMC Biotechnol. 2023 Aug 23;23:31. doi: 10.1186/s12896-023-00804-7 (PMC10463907; doi:10.1186/s12896-023-00804-7)
Supplement: Supplementary file 1 — Supplementary Material 1 [file 12896_2023_804_MOESM1_ESM.docx]

**Supplementary table 1:** Microbial and antibiotic resistance gene target sequences used for molecular inversion probe design. The 5 nt pathogen ID is highlighted in bold. Probe arm sequences are highlighted in green.

| No | Target | Probe name | Accession number | Target sequence |
| --- | --- | --- | --- | --- |
| 1 | Aspergillus fumigatus | MIP_Afum_1 | XM_750992.1 | 5’-**TTCTC**AACGGGGGTGTTGCGTACACTCGTACGGTGGACCGGGTTGGA-3’ |
| 2 | Aspergillus fumigatus | MIP_Afum_2 | L76086.1 | 5’-**TTCTC**CATCGCTGGCTAGGACCCCCTAGGTCGATTACCTCCGATCGCCT-3’ |
| 3 | Aspergillus fumigatus | MIP_Afum_3 | AY433801.1 | 5’-**TTCTC**AAGAAGTATGACGCTTTACCTAGTCTACGGGAATTGCTAACAGATCCAGC-3’ |
| 4 | Candida albicans | MIP_Calb_1 | CP025158.1 | 5’-**TTGCT**ACTGACTCAAGTAAAGAACGGAGTGCAATTTAAAGTAATTGGCCCGTTA-3’ |
| 5 | Candida albicans | MIP_Calb_2 | CP025165.1 | 5’-**TTGCT**AGAACTCATAATCCAAAGGTATAAATCGGCACTAGTTTGATTTTTGTAGCGT-3’ |
| 6 | Candida albicans | MIP_Calb_3 | CP025163.1 | 5’-**TTGCT**GCATTTGTTGATATTGCTGCAAATTAGAGCAGTAAGTTAGATCATTTTGAATT-3’ |
| 7 | Candida albicans | MIP_Calb_4 | CP025165.1 | 5’-**TTGCT**GGCCTCATAAGCCGCAATCTTTGTGACCGTCGTACTTAAACCACTTGAAC-3’ |
| 8 | Candida albicans | MIP_Calb_5 | CP025158.1 | 5’-**TTGCT**CAGTGCATTTAGTATACTGAGTGAACTGGTACCTGTGACATTCAAGATAACTG-3’ |
| 9 | Candida albicans | MIP_Calb_6 | CP025163.1 | 5’-**TTGCT**ACTGAGAAACCTTTGGTGGAGCATAACTATGACTGGTCGTTGCAGTAT-3’ |
| 10 | Candida albicans | MIP_Calb_7 | CP025159.1 | 5’**-TTGCT**TGCCGGTTGCCAACGCTTGTACCATTCTCTAGTACCAACTTACATCCT-3’ |
| 11 | Candida albicans | MIP_Calb_8 | CP025182.1 | 5’-**TTGCT**GATCTGCCATTATTGTACTGGCTTGATCTAGACTAATGAAAGTGTTTCCTGTA-3’ |
| 12 | Candida albicans | MIP_Calb_9 | CP025165.1 | 5’-**TTGCT**TCTTAATACAATAATTTAATACGGTAGTTAGTTGGTTTTCACTAATCAATTAACT-3’ |
| 13 | Candida albicans | MIP_Calb_10 | CP025152.1 | 5’-**TTGCT**ATAAACCTATCGACCCTAGATCACGAGCACAGAAACGTGCTAAAAGCTATT-3’ |
| 14 | Candida albicans | MIP_Calb_11 | CP025165.1 | 5’-**TTGCT**CTTTTATACCAAACTCATGTTTCACGTCCTTCCGGCATATGTTTCC-3’ |
| 15 | Candida dubliniensis | MIP_Cdub_1 | FM992690.1 | 5’-**TTAGG**ACGGCTAGTGGTACACTAGGAGTAGCGCCAAACGCGTCTTCAC-3’ |
| 16 | Candida dubliniensis | MIP_Cdub_2 | FM992693.1 | 5’-**TTAGG**ATTAGTAATGGGCCTATAGGCACGTCGAAACTTATATTCCCCAACGACAA-3’ |
| 17 | Candida dubliniensis | MIP_Cdub_3 | FM992689.1 | 5’-**TTAGG**ATATGGGGAGGTCCGCTACTCGTGTAAGGGAATTCTGTATTTTGAAAAAC-3’ |
| 18 | Candida Glabrata | MIP_Cgla_1 | CR380956.2 | 5’-**TCTTG**TCAGAACCTGACTCCATACTAACATACTCGTCCGCACTCACACG-3’ |
| 19 | Candida Glabrata | MIP_Cgla_2 | CR380951.2 | 5’-**TCTTG**GTGATATCTACCTGGTACACGTAATCTAACGAACATAGCATGTTGAAC-3’ |
| 20 | Candida Glabrata | MIP_Cgla_3 | CR380951.2 | 5’-**TCTTG**CTTTAATGGTCAGAGCGCTAGTGCGTACATCGGTTCATACCCCTATTG-3’ |
| 21 | Citrobacter koseri | MIP_Ckos_1 | CP000822.1 | 5’-**TCCGT**TAGCCGGGTTTAAGCGTCAACTAAGCCCGTAATTTGCGATACACGAGAA-3’ |
| 22 | Citrobacter koseri | MIP_Ckos_2 | CP000822.1 | 5’-**TCCGT**CGTTACCGGCATTCCGACGTAAGGGGCGATTTCCGCCAGAT-3’ |
| 23 | Candida parapsilosis | MIP_Cpar_1 | HE605203.1 | 5’-**TCGAC**TCTACAGTCTCTCGTGTCTACCCCGCGTGAGTGGAGACGGTGCG-3’ |
| 24 | Candida parapsilosis | MIP_Cpar_2 | HE605204.1 | 5’-**TCGAC**GATGCCCACTTACGTGACTAGACTCCCCATACCGACGCGTCAG-3’ |
| 25 | Candida parapsilosis | MIP_Cpar_3 | HE605206.1 | 5’-**TCGAC**CAACCAAACCATGCCTATCCAACTAACGGACGATGAGCCTTTCACG-3’ |
| 26 | Candida tropicalis | MIP_Ctro_1 | XM_002548020.1 | 5’-**TCACA**CACATAAAGCACCCTACCGGTTAACGTGGGAGGAGAAGCAAGAATTAGA-3’ |
| 27 | Candida tropicalis | MIP_Ctro_2 | XM_002548636.1 | 5’-**TCACA**GGTAATGACAATCTTGTATGCATATGGGACGCTAGATCTACGTCTAATGCTC-3’ |
| 28 | Candida tropicalis | MIP_Ctro_3 | XM_002546864.1 | 5’-**TCACA**AATGATAAGTTTGTGGCCTCCGATAGGGTTGGTCATTTCTGGAATTTTTTAC-3’ |
| 29 | Escherichia coli | MIP_Ecol_1 | CP024278.1 | 5’-**TGTCC**GGTGATGCGCATTATGTGTCGTCGCGGCTTCGAAATGGACTTTGCTG-3’ |
| 30 | Escherichia coli | MIP_Ecol_2 | CP024278.1 | 5’-**TGTCC**AACGTCGTTACTATCCTTACGGTTCGGCGTTGACCCACGTCATCG-3’ |
| 31 | Escherichia coli | MIP_Ecol_3 | CP024278.1 | 5’-**TGTCC**AAGCCAGCCAGTTCCAGGATTTTCGCTTCCCACTCTGCGTCG-3’ |
| 32 | Escherichia coli | MIP_Ecol_4 | CP024278.1 | 5’-**TGTCC**GAGGAAGGGAGTAAAGTTAATACCTTTGCTCATTGACGTTACCCGCA-3’ |
| 33 | Enterococcus faecalis | MIP_Efae_1 | CP008816.1 | 5’-**TGCAG**GATCGCTTTGTTGGCTTAATCGGCTTGACAGACGTTGTAGATAAAATGTTTGATT-3’ |
| 34 | Enterococcus faecalis | MIP_Efae_2 | CP008816.1 | 5’-**TGCAG**CTGATTTAATCACGCCAGATTCGCCGACACAACGTGTAGGTGGCAA-3’ |
| 35 | Enterococcus faecalis | MIP_Efae_3 | CP008816.1 | 5’-**TGCAG**TTAGAAGTTGTTGAGATCGGAGCAACGCGTAGTGTTGGTTCTGTTCCC-3’ |
| 36 | Enterococcus faecalis | MIP_Efae_4 | CP008816.1 | 5’-**TGCAG**GGACTTCTTACGGACTATTAGGAATCCCAGGAGACTTC-3’ |
| 37 | Enterococcus faecalis | MIP_Efae_5 | CP008816.1 | 5’-**TGCAG**CTAAAGTCATTCGGCGGGCGCTAGACATTTGGAAACCAATCGG-3’ |
| 38 | Enterococcus faecalis | MIP_Efae_6 | CP029612.1 | 5’-**TGCAG**TATGCCATGCGGCATAAACTGTTATGCGGTATTAGCACCTGTTTCCAAGT-3’ |
| 39 | Enterococcus faecalis | MIP_Efae_7 | CP008816.1 | 5’-**TGCAG**GAATTTACAGATCCGGAACAAATTTTTCACGAGATTCGGATGAATTGG-3’ |
| 40 | Enterococcus faecalis | MIP_Efae_8 | CP008816.1 | 5’-**TGCAG**TGGTTCCCATTTTCTTCGTAATACTCCACGTAATCCCAACTGGCAAGA-3’ |
| 41 | Enterococcus faecalis | MIP_Efae_9 | CP008816.1 | 5’-**TGCAG**GAATTTACCGTTTAGTGTGCATACGAGAGAGGCATTAGAAGATACCTATCGA-3’ |
| 42 | Enterococcus faecalis | MIP_Efae_10 | CP029612.1 | 5’-**TGCAG**AAACTAATGGCACTCGTAGTACCGACCGCATTTTTCAACTCGGTAGGTTG-3’ |
| 43 | Enterococcus faecalis | MIP_Efae_11 | CP008816.1 | 5’-**TGCAG**TACCGACCGCATTTTTCAACTCGGTAGGTTGGTTGTAATTTGAGAAACTCG-3’ |
| 44 | Enterococcus faecalis | MIP_Efae_12 | CP008816.1 | 5’-**TGCAG**CTATCAGTTACCGGATTTGTAACGTGTCGGACAGAATACTTTTGTGGAGAAGG-3’ |
| 45 | Enterococcus faecium | MIP_Efam_1 | CP014449.1 | 5’-**TGGTA**CTTGTAATTTCCCTTTACCGATAATCGTTTGCCGATCCACTTGTG-3’ |
| 46 | Enterococcus faecium | MIP_Efam_2 | CP014449.1 | 5’-**TGGTA**TGCTTCCGCCGTTTATACGTGTTTAGAGCGTGAGACAAAAGTGTTCTTTAC-3’ |
| 47 | Enterococcus faecium | MIP_Efam_3 | CP018065.1 | 5’-**TGGTA**CAAAACGAACGGGGAATGGCGATACTTTGACAGCACAAGAGTGGCT-3’ |
| 48 | Enterococcus faecium | MIP_Efam_4 | CP014449.1 | 5’-**TGGTA**AATACTGTAGACATTTTCGCTTGCTACACCGACTTCTTTTGGTTCCATGT-3’ |
| 49 | Enterococcus faecium | MIP_Efam_5 | CP014449.1 | 5’-**TGGTA**ATGAGATCAATGCGTAACTGACGCTAAGTGCAACATGCCCCAGCGAGG-3’ |
| 50 | Enterococcus faecium | MIP_Efam_6 | CP014449.1 | 5’-**TGGTA**TAAATGAGATCAATGCGTAACTGACGCTAAGTGCAACATGCCCCAG-3’ |
| 51 | Enterococcus faecium | MIP_Efam_7 | CP011281.1 | 5’-**TGGTA**AGATGCGGTTGTTTTATACGGAAATTCGCCAGAAATCGGGAAAGTAGAAGA-3’ |
| 52 | Enterococcus faecium | MIP_Efam_8 | CP018065.1 | 5’-**TGGTA**CTTTTTGGACCGCTTCTGGCGAAATCTCGTCCCCAATCTTTTTTTTGA-3’ |
| 53 | Haemophilus influenzae | MIP_Hinf_1 | FQ670204.1 | 5’-**CTTCG**ACACCTTTCGCTTCACTCACTCTCAGAGTGGTGGGTCTGAGTGG-3’ |
| 54 | Haemophilus influenzae | MIP_Hinf_2 | FQ670204.1 | 5’-**CTTCG**GCTTAGTACCACAATATGGTTTTTAGATACGGGATTATCACCCTCTTTGA-3’ |
| 55 | Haemophilus influenzae | MIP_Hinf_3 | CP020014. | 5’-**CTTCG**CTTTCGTTTCACTCACTCTCAGAGTGGTGGGTCTGAGTGGACTTGAACC-3’ |
| 56 | Klebsiella oxytoca | MIP_Koxy_1 | CP029128.1 | 5’-**CTCGA**GGCAGAAGTGCATAGATGCACGCTTCATTACGGCGCATGATTATG-3’ |
| 57 | Klebsiella pneumoniae | MIP_Kpne_1 | CP022611.1 | 5’-**CTAAC**GGGAGAAGGCACGCTGGTGTGTAGGTGAAGTCCCTGCGGATG-3’ |
| 58 | Klebsiella pneumoniae | MIP_Kpne_2 | CP022611.1 | 5’-**CTAAC**TCAGAGTCAACCCGTTTTTCAGGATTTTTCTCTGCAACCGAACCG-3’ |
| 59 | Klebsiella pneumoniae | MIP_Kpne_3 | CP022611.1 | 5’-**CTAAC**TCCGTGCAAAACACGGCCCTCAGCCCGCATCCATGCGGGCTG-3’ |
| 60 | Klebsiella pneumoniae | MIP_Kpne_4 | CP022611.1 | 5’-**CTAAC**CCTGAACCATAACCCGAACAATCCGGCCTGGGCTGACCGTGAC-3’ |
| 61 | Klebsiella pneumoniae | MIP_Kpne_5 | CP022611.1 | 5’-**CTAAC**CGCAGGGACTTCACCTACACACCAGCGTGCCTTCTCCCGAAG-3’ |
| 62 | Klebsiella pneumoniae | MIP_Kpne_6 | CP022611.1 | 5’-**CTAAC**ACTTTACCCGGCCGAGTATCGTTATCAAACCTAACGCCAATGC-3’ |
| 63 | Klebsiella pneumoniae | MIP_Kpne_7 | CP022611.1 | 5’-**CTAAC**GCAGGGACTTCACCTACACACCAGCGTGCCTTCTCCCGAAGTTAC-3’ |
| 64 | Klebsiella pneumoniae | MIP_Kpne_8 | CP022611.1 | 5’**-CTAAC**GGCTGGCCACCTGGATACCTATATCCCGGAACCAGAGCGT-3’ |
| 65 | MecA antibiotic resistance gene | MIP_mecA_1 | CP030326.1 | 5’-**CCTAT**GCTATTATCGTCAACGATTGTGACACGATAGCCATCTTCATGTTGGAG-3’ |
| 66 | MecA antibiotic resistance gene | MIP_mecA_2 | CP030326.1 | 5’**-CCTAT**AGCAACCATCGTTACGGATTGCTTCACTGTTTTGTTATTCATCTATATCGTAT-3’ |
| 67 | MecA antibiotic resistance gene | MIP_mecA_3 | CP030326.1 | 5’-**CCTAT**TGTATTGGCCAATTCCACATTGTTTCGGTCTAAAATTTTACCACGTTCTGATTT-3’ |
| 68 | MecA antibiotic resistance gene | MIP_mecA_4 | NG_047955.1 | 5’-**CGCTT**GCCAAAAAATTTGAGCAAGGTATGCAAGATTTGGGAATCGGTGAAAA-3’ |
| 69 | MecA antibiotic resistance gene | MIP_mecA_5 | NG_047955.1 | 5’-**CGCTT**AATCGGTGAAAATATCCCGAGTGATTATCCCTTTTATAAAGCACAAATCTCA-3’ |
| 70 | MecA antibiotic resistance gene | MIP_mecA_6 | HF569116.1 | 5’-**CGCTT**GGATAATCACTCGGGATATTTTCACCGATTCCCAAATCTTGCATACCTT-3’ |
| 71 | Proteus mirabilis | MIP_Pmirab_1 | CP017085.1 | 5’-**CATTC**GGTCGGGGTTGTCTGGCTCTTGCGAGGTTAACCGCAACATATAGTGTTTA-3’ |
| 72 | Neisseria meningitidis | MIP_Nmen_1 | CP015886.1 | 5’-**CAGCA**GCCGTTGCTTTTGTCTTGGCGAAGGTAGCGGTAAATGGTGCTGTGGTG-3’ |
| 73 | Neisseria meningitidis | MIP_Nmen_2 | CP015886.1 | 5’-**CAGCA**GTTCAGTTGCTACGGTTACTGTCAGGTTTCGGTTATGTTGGAATTTCG-3’ |
| 74 | Neisseria meningitidis | MIP_Nmen_3 | CP007668.1 | 5’-**CAGCA**TCCACCACAACGGTATAGACCCGTCCGTTGCGTTTCAGAATGCC-3’ |
| 75 | Pseudomonas aeruginosa | MIP_Paer_1 | CP015117.1 | 5’-**CAAGT**CGTGTGTCTCCCATGCTCGGCACTTCTGGGTATTCGGAGTTTGCA-3’ |
| 76 | Pseudomonas aeruginosa | MIP_Paer_2 | CP017149.1 | 5’-**CAAGT**TCAAGCATTTACTCTTCGTCGTCGCCGGGAGTGGTGCGCATTATAGG-3’ |
| 77 | Pseudomonas aeruginosa | MIP_Paer_3 | CP017149.1 | 5’-**CAAGT**GTGCGGCACGTGACGCTGGCCTACACCCTCTTCGATATCACTGC-3’ |
| 78 | Pseudomonas aeruginosa | MIP_Paer_4 | CP011857.1 | 5’-**CAAGT**CAGGAACAGATCTTCGTCCATGCCCAGCGCGACTGGGACGAGAACAT-3’ |
| 79 | Pseudomonas aeruginosa | MIP_Paer_5 | CP017149.1 | 5’-**CAAGT**TGGCCTTCCTGGTAGGTGTTGCCGACCGAATCCTCGGTGAAGGC-3’ |
| 80 | Pseudomonas aeruginosa | MIP_Paer_6 | CP017149.1 | 5’-**CAAGT**GGTGCCGGTGATGGACATATAGGCGGGGGTTGCCATGGATAC-3’ |
| 81 | Pseudomonas aeruginosa | MIP_Paer_7 | CP017149.1 | 5’-**CAAGT**AACCAGGGAATCACGGCACACATGAGCAAGACGATCACATGGACG-3’ |
| 82 | Pseudomonas aeruginosa | MIP_Paer_8 | CP017149.1 | 5’-**CAAGT**CGATGAGGAACGACAGGGCGAGCATCTTCAGCGAGGGGTGTTT-3’ |
| 83 | Pseudomonas aeruginosa | MIP_Paer_9 | CP017149.1 | 5’-**CAAGT**TGGCATCGGTCAATGCCGCTAGTCGGCCTTCCTTCTGCCATTGG-3’ |
| 84 | Streptococcus agalactiae | MIP_Saga_1 | CP019814.1 | 5’-**GTTGT**GTCACGGAAACAAAGGTGTCGTCTCACGTATTGTTCCTGTTGAAGATATGC-3’ |
| 85 | Streptococcus agalactiae | MIP_Saga_2 | CP030845.1 | 5’-**GTTGT**GTAAGAGTTGCACTTGACGGATATCTAGGCGATCAAATAAACAAGCAAA-3’ |
| 86 | Streptococcus agalactiae | MIP_Saga_3 | CP019837.1 | 5’-**GTTGT**CCCGATCATATTGGTCCTATCGAGCACCCTACATTCGAAGATTATTTTTTC-3’ |
| 87 | Staphylococcus aureus | MIP_Saur_1 | CP030326.1 | 5’-**GTGTG**CATTAGGGACAGTACCTAAGTTAGACGATTCTCGTGTCTTACTTGCACAAG-3’ |
| 88 | Staphylococcus aureus | MIP_Saur_2 | CP030326.1 | 5’-**GTGTG**TTTTAAAAGATTAGCGTTATACGGTGAGTACAACATGATCTGTTAATATAACAAGCC-3’ |
| 89 | Staphylococcus aureus | MIP_Saur_3 | CP030326.1 | 5’-**GTGTG**CTTAGCGTCGATGGTAGTCGAACTTACGTTCCGCTAGAGTAGAACGTT-3’ |
| 90 | Staphylococcus aureus | MIP_Saur_4 | CP030326.1 | 5’-**GTGTG**ATAAATCAATGTTCTATGCTCTACGAAGTTATATTGGCAGTAGTTGACTGAAC-3’ |
| 91 | Staphylococcus aureus | MIP_Saur_5 | CP030326.1 | 5’-**GTGTG**GGTAATAATCCTGTAGTCGAAAATGTTGTCTCTCTTGAGTGGATCCTGAG-3’ |
| 92 | Staphylococcus aureus | MIP_Saur_6 | CP030326.1 | 5’-**GTGTG**AGGATAGGCGAAGCGTGCGATTGGATTGCACGTCTAAGCAGTAAGG-3’ |
| 93 | Staphylococcus epidermidis | MIP_Sepi_1 | CP010942.1 | 5’-**GCCTA**GTACGGTTTAACATTAATTCTGAATCGTGAGGGTTAACTCGTAAGTCAGCACG-3’ |
| 94 | Staphylococcus epidermidis | MIP_Sepi_2 | CP022247.1 | 5’-**GCCTA**ATCGGTCGTGTAGCACAAGTGCATATTGACGATAAAGTAATTATGGACAACG-3’ |
| 95 | Staphylococcus haemolyticus | MIP_Shae_1 | AP006716.1 | 5’-**GCAAG**ACTTAAGTGAGTGGGTTATCTATCTAAGATCGACTTTAGAGAACTATTTTAGAAC-3’ |
| 96 | Staphylococcus haemolyticus | MIP_Shae_2 | CP013911.1 | 5’-**GCAAG**GTTCGATGGCTAATCCGTAAGTGACATCGGCATTGCCTACTGCTTTA-3’ |
| 97 | Staphylococcus haemolyticus | MIP_Shae_3 | CP013911.1 | 5’-**GCAAG**TACAAGTATCCTCGTCTCTTCGATATTGTACACAAGGCCATCGATCAGAC-3’ |
| 98 | Staphylococcus lugdunensis | MIP_Slug_1 | LS483312.1 | 5’-**GGTAA**GCTTGAGTATTGCACTTTGACTAATACGTCACCTTCTGGTAGGTCGTCG-3’ |
| 99 | Staphylococcus lugdunensis | MIP_Slug_2 | CP020769.1 | 5’-**GGTAA**TGTCAGTGTGATCAGCGTTAGGCTGCTCATTCGTTTCCACTGTCGG-3’ |
| 100 | Staphylococcus lugdunensis | MIP_Slug_3 | LS483312.1 | 5’-**GGTAA**ACGTATTACCACTGCTATACATAGCGTACAAAATACGGCGTTGTACTGG-3’ |
| 101 | Streptococcus mutans | MIP_Smut_1 | CP013237.1 | 5’-**GGATC**ACATGCTTTAAAACGAACCCTAGCAGAGGCCGCTGCTATTAGTCAGGTGA-3’ |
| 102 | Streptococcus mutans | MIP_Smut_2 | CP013237.1 | 5’-**GGATC**GAAGTCATGCGGACACTTAATACGCTGGATGTTACCGCCATAGAAGAAA-3’ |
| 103 | Streptococcus pneumoniae | MIP_Spne_1 | CP000410.2 | 5’-**GACCT**GTGGAAAGAGATTCGTAAACGTGGATTTAAGAATAAAGCCTTTCGAACTTT-3’ |
| 104 | Streptococcus pneumoniae | MIP_Spne_2 | CP000410.2 | 5’-**GACCT**GCTCTTAGTACTTGGTTTGTTTACCGTTATGCGGATAAGATTCAAAAAGAT-3’ |
| 105 | Streptococcus pneumoniae | MIP_Spne_3 | CP000410.2 | 5’-**GACCT**AGGGTTATACTCCTGGTCACAAATCCTACGTCAGTCAAGAGAAGATTGC-3’ |
| 106 | Streptococcus pneumoniae | MIP_Spne_4 | CP000410.2 | 5’-**GACCT**GTCTTTGTCACTTGCTTCTCTCAAGCGACAACTATATTAGTATATCACAGCCA-3’ |
| 107 | Streptococcus pneumoniae | MIP_Spne_5 | CP000410.2 | 5’-**GACCT**AACTTTTAAGTTAATTCGGGCATCCAACTCTTGTTCTGGTTGGAAGTACTTTTC-3’ |
| 108 | Streptococcus pyogenes | MIP_Spyo_1 | CP021640.1 | 5’-**ATGGC**TCGGGGCATTACTGACAACAGGTAGCGTTATTGTTACCTGGCTTGGAG-3’ |
| 109 | Streptococcus pyogenes | MIP_Spyo_2 | CP021640.1 | 5’**-ATGGC**CTAGTGTATAGCCATATACGTTAGCATTGACTGCTGTTCCATTTGAAAAG-3’ |
| 110 | Streptococcus pyogenes | MIP_Spyo_3 | CP021640.1 | 5’-**ATGGC**GCTTTTTTCAGAGATATAGGAGGTTTGACTAGCGATGCCAACATTTCTA-3’ |
| 111 | Streptococcus salivarius | MIP_Ssal_1 | LS483366.1 | 5’-**ACTGA**GAGTGCTAGGTGTTGGATCCTTTCCGGGATTCAGTGCCGCAGCTA-3’ |
| 112 | Streptococcus salivarius | MIP_Ssal_2 | LS483366.1 | 5’-**ACTGA**CTATGACCTCGGGACGCCAGTTTCGAGTGAGGCGTTGTTGGG-3’ |
| 113 | Streptococcus salivarius | MIP_Ssal_3 | CP014144.1 | 5’-**ACTGA**CACTGCGGCTGACCATAAGTCAGCGCCCCTTCTCCCGAAGTTACG-3’ |
| 114 | Streptococcus sanguinis | MIP_Ssang_1 | CP000387.1 | 5’-**ACGTT**GTCCCAAGAACCGTTCCCTTACTAGGCCTTTAAGCCTATCCAGGTCTTC-3’ |
| 115 | Streptococcus sanguinis | MIP_Ssang_2 | CP000387.1 | 5’-**ACGTT**GTTCCATCTGATCAACGATGTCGTATGGGCTAGTGTAAGTCTGATTGTCAATC-3’ |
| 116 | Staphylococcus saprophyticus | MIP_Ssapr_1 | AP008934.1 | 5’-**AGCCA**TCTAGGACTGTCGGCTATCTTATCCGGACTCGTCATCTTGCCCGGA-3’ |
| 117 | Staphylococcus saprophyticus | MIP_Ssapr_2 | AP008934.1 | 5’-**AGCCA**TCAAATATCGCATTATAAGTTTGACGTGTCCGTAATAAATCCGAAATATAAATAT-3’ |
| 118 | Staphylococcus aureus | MIP_Staph_1 | CP030323.1 | 5’-**AACTG**CCTTCCTCTGGAAACCTTAGTCAATCGGTGGACGGGATTCTCACC-3’ |
| 119 | Staphylococcus aureus | MIP_Staph_2 | CP030323.1 | 5’-**AACTG**GAATTTCACGTGCTCCGTCGTACTCAGGATCCACTCAAGAGAGACAACATT-3’ |
| 120 | Staphylococcus aureus | MIP_Staph_3 | CP030323.1 | 5’-**AACTG**TCTCCGCTACCCTCAGTTCATCCGCTCACTTTTCAACGTAAGTCGGTT-3’ |
| 121 | Streptococcus sp. | MIP_Strep_1 | LS483335.1 | 5’-**TGCAC**CGTGTGCCTACAACAAGTTCGAGCCCGTTAATGGGTGAGAGCGTG-3’ |
| 122 | Streptococcus sp. | MIP_Strep_2 | LS483520.1 | 5’-**TGCAC**TGGGTTAGTCGGGACCTAAGGAGAGACCGAAAGGTGTATCCGATG-3’ |
| 123 | Streptococcus sp. | MIP_Strep_3 | LS483334.1 | 5’-**TGCAC**ATTTGTAATCAGAGGGTCGCGTGTTCAAGTCATGTAGCCGGCATT-3’ |
| 124 | Streptococcus sp. | MIP_Strep_4 | MH628246.1 | 5’-**TGCAC**CAGCCGCGGTAATACGTAGGTCCCGAGCGTTGTCCGGATTTATTG-3’ |
| 125 | Streptococcus sp. | MIP_Strep_5 | LS483334.1 | 5’-**TGCAC**CCGTAGTGTAGCGGTTATCACGTCGCCCTGTCACGGCGAAGATC-3’ |
| 126 | Streptococcus sp. | MIP_Strep_6 | LS483335.1 | 5’-**TGCAC**GTCCACCGTAAGACTAAGGTTTCCAGGGGAAGGCTCGTCCGCC-3’ |
| 127 | Streptococcus sp. | MIP_Strep_7 | LS483335.1 | 5’-**TGCAC**AGTCAGACTGCGAGTGCTAAGATCCGTAGTCGAAAGGGAAACAGC-3’ |
| 128 | VanA gene | MIP_vanA_1 | CP025391.1 | 5’-**GTGCT**CAGAGGAGCGAGGACGGATACAGGAAACGGCAAAAAAAATATATAAAGC-3’ |
| 129 | VanA gene | MIP_vanA_2 | KX574671.1 | 5’-**GTGCT**TTTCCGGCTCGACTTCCTGATGAATACGAAAGATTCCGTACTGCAG-3’ |
| 130 | VanA gene | MIP_vanA_3 | MG674582.1 | 5’-**GTGCT**GGAAACAGTGCCGCGTTAGTTGTTGGCGAGGTGGACCAAATCAGG-3’ |
| 131 | Escherichia coli | MIP_EC1 | CP119120.1 | 5’-**TGTCC**AGTCGAGCATCTCTTCAGCGTAAGGGTAATGCGAGGTACG-3’ |
| 132 | Klebsiella oxytoca | MIP_Koxy2 | OX030698.1 | 5’-**CTCGA**CCTGCTGCAGCGCCTGGGTATTCAGCGTGGTGCCGTCAC-3’ |
| 133 | Klebsiella pneumoniae | MIP_Kpneu2 | CP090222.1 | 5’-**CTAAC**ATTCACAAGAAGCCGTTGATGTGAAACCTGGGTCAAAGGTA-3’ |
| 134 | Pseudomonas aeruginosa | MIP_PA3 | CP117300.1 | 5’-**CAAGT**CGACCTGTCGACCACCGGCAGTCCGGCGATTTCCAGCCAGTAC-3’ |
| 135 | Pseudomonas aeruginosa | MIP_PA5 | CP117300.1 | 5’-**CAAGT**GAACACCTTGATGTTCGAAGGCTTCTCGTGGCCGATCGGCACC-3’ |
| 136 | Pseudomonas aeruginosa | MIP_PA6 | MK264344.1 | 5’-**CAAGT**CACTTTCTCCCTCAGGACGTATGCGGTATTAGCGCC-3’ |
| 137 | Proteus mirabilis | MIP_Pmira1 | CP046048.1 | 5’-**CATTC**GGGGCAACCACCGCTAAGTTGGTAGTTTGAGAATGCGCA-3’ |
| 138 | Proteus mirabilis | MIP_Pmira2 | CP046048.1 | 5’-**CATTC**ACGACGACGTAATGCTAATAAGTAGCTACCTTTAGCAAA-3’ |
| 139 | Staphylococcus aureus | MIP_SA2 | CP119113.1 | 5’-**GTGTG**ACCTGAAACAAAGCATCCTAAAAAAGGTGTAGAGAAATATGG-3’ |
| 140 | MecA gene | MIP_mecA1 | OP104250.1 | 5’-**CCTAT**TGAATTATTAGCACTTGTAAGCACACCTTCATATGACGTCTAT-3’ |
| 141 | Klebsiella pneumoniae carbapenem resistant beta-lactamase | MIP_KPC | ON921318.1 | 5’-**GCATT**TACAGTGATAACGCCGCCGCCAATTTGTTGCTGAAGGAGTTGG-3’ |
| 142 | New Delhi metallo-beta-lactamase | MIP_NDM2 | NG_076661.1 | 5’-**ATCGC**CACCGAATGTCTGGCAGCACACTTCCTATCTCGACATGCCGG-3’ |
| 143 | OXA-48 beta-lactamase | MIP_Oxa_48 | CP104800.1 | 5’-**GTAGA**AAGGATTTACCAATAATCTTAAACGGGCGAACCAAGCATTTTTA-3’ |
| 144 | VIM beta-lactamase | MIP_VIM | ON059758.1 | 5’-**TCCAT**GGTGATGAGTTGCTTTTGATTGATACAGCGTGGGGTGCG-3’ |
