# Supplementary material for: Highly multiplexed targeted sequencing strategy for infectious disease surveillance
Source: BMC Biotechnol. 2023 Aug 23;23:31. doi: 10.1186/s12896-023-00804-7 (PMC10463907; doi:10.1186/s12896-023-00804-7)
Supplement: Supplementary file 2 — Supplementary Material 2 [file 12896_2023_804_MOESM2_ESM.docx]

**Supplementary table 2:** List of molecular inversion probe sequences. The sequences that correspond to the complementary to the target MIP arms are highlighted with blue. The 5 nt pathogen ID is highlighted with red, forward and reverse primers are highlighted with green and yellow respectively and the 9 nt UMI (NNNNNNNNN) sequence part is highlighted with black.

|  | MIP per target | Molecular inversion probe sequence |
| --- | --- | --- |
| 1 | BMGF_MIP_Afum_1 | 5’-TACGCAACACCCCCGTTGAGAACTTCAGCTTCCCGATATCCGACGGTAGTGTNNNNNNNNNTCCAACCCGGTCCAC-3’ |
| 2 | BMGF_MIP_Afum_2 | 5’-GGTCCTAGCCAGCGATGGAGAACTTCAGCTTCCCGATATCCGACGGTAGTGTNNNNNNNNNAGGCGATCGGAGGTAAT-3’ |
| 3 | BMGF_MIP_Afum_3 | 5’-GGTAAAGCGTCATACTTCTTGAGAACTTCAGCTTCCCGATATCCGACGGTAGTGTNNNNNNNNNGCTGGATCTGTTAGCAATTC-3’ |
| 4 | BMGF_MIP_Calb_1 | 5’-CGTTCTTTACTTGAGTCAGTAGCAACTTCAGCTTCCCGATATCCGACGGTAGTGTNNNNNNNNNTAACGGGCCAATTACTTTA-3’ |
| 5 | BMGF_MIP_Calb_2 | 5’-ACCTTTGGATTATGAGTTCTAGCAACTTCAGCTTCCCGATATCCGACGGTAGTGTNNNNNNNNNACGCTACAAAAATCAAACTAGT-3’ |
| 6 | BMGF_MIP_Calb_3 | 5’-GCAGCAATATCAACAAATGCAGCAACTTCAGCTTCCCGATATCCGACGGTAGTGTNNNNNNNNNAATTCAAAATGATCTAACTTACT-3’ |
| 7 | BMGF_MIP_Calb_4 | 5’-ATTGCGGCTTATGAGGCCAGCAACTTCAGCTTCCCGATATCCGACGGTAGTGTNNNNNNNNNGTTCAAGTGGTTTAAGTACGAC-3’ |
| 8 | BMGF_MIP_Calb_5 | 5’-CTCAGTATACTAAATGCACTGAGCAACTTCAGCTTCCCGATATCCGACGGTAGTGTNNNNNNNNNCAGTTATCTTGAATGTCACAGG-3’ |
| 9 | BMGF_MIP_Calb_6 | 5’-CCACCAAAGGTTTCTCAGTAGCAACTTCAGCTTCCCGATATCCGACGGTAGTGTNNNNNNNNNATACTGCAACGACCAGTCA-3’ |
| 10 | BMGF_MIP_Calb_7 | 5’-GCGTTGGCAACCGGCAAGCAACTTCAGCTTCCCGATATCCGACGGTAGTGTNNNNNNNNNAGGATGTAAGTTGGTACTAGAG-3’ |
| 11 | BMGF_MIP_Calb_8 | 5’-CAGTACAATAATGGCAGATCAGCAACTTCAGCTTCCCGATATCCGACGGTAGTGTNNNNNNNNNTACAGGAAACACTTTCATTAGTC-3’ |
| 12 | BMGF_MIP_Calb_9 | 5’-ATTAAATTATTGTATTAAGAAGCAACTTCAGCTTCCCGATATCCGACGGTAGTGTNNNNNNNNNAGTTAATTGATTAGTGAAAACCAAC-3’ |
| 13 | BMGF_MIP_Calb_10 | 5’-CTAGGGTCGATAGGTTTATGAGAACTTCAGCTTCCCGATATCCGACGGTAGTGTNNNNNNNNNAATAGCTTTTAGCACGTTTCTG-3’ |
| 14 | BMGF_MIP_Calb_11 | 5’-ACATGAGTTTGGTATAAAAGGAGAACTTCAGCTTCCCGATATCCGACGGTAGTGTNNNNNNNNNGGAAACATATGCCGGA-3’ |
| 15 | BMGF_MIP_Cdub_1 | 5’-TAGTGTACCACTAGCCGTCCTAACTTCAGCTTCCCGATATCCGACGGTAGTGTNNNNNNNNNTGTGAAGACGCGTTTG-3’ |
| 16 | BMGF_MIP_Cdub_2 | 5’-CCTATAGGCCCATTACTAATCCTAACTTCAGCTTCCCGATATCCGACGGTAGTGTNNNNNNNNNTTGTCGTTGGGGAATATAAG-3’ |
| 17 | BMGF_MIP_Cdub_3 | 5’-AGCGGACCTCCCCATATCCTAACTTCAGCTTCCCGATATCCGACGGTAGTGTNNNNNNNNNGTTTTTCAAAATACAGAATTCCC-3’ |
| 18 | BMGF_MIP_Cgla_1 | 5’-GTATGGAGTCAGGTTCTGACAAGACTTCAGCTTCCCGATATCCGACGGTAGTGTNNNNNNNNNCGTGTGAGTGCGGAC-3’ |
| 19 | BMGF_MIP_Cgla_2 | 5’-TGTACCAGGTAGATATCACCAAGACTTCAGCTTCCCGATATCCGACGGTAGTGTNNNNNNNNNGTTCAACATGCTATGTTCG-3’ |
| 20 | BMGF_MIP_Cgla_3 | 5’-AGCGCTCTGACCATTAAAGCAAGACTTCAGCTTCCCGATATCCGACGGTAGTGTNNNNNNNNNCAATAGGGGTATGAACCGA-3’ |
| 21 | BMGF_MIP_Ckos_1 | 5’-GACGCTTAAACCCGGCTAACGGACTTCAGCTTCCCGATATCCGACGGTAGTGTNNNNNNNNNTTCTCGTGTATCGCAAATTAC-3’ |
| 22 | BMGF_MIP_Ckos_2 | 5’-CGGAATGCCGGTAACGACGGACTTCAGCTTCCCGATATCCGACGGTAGTGTNNNNNNNNNATCTGGCGGAAATCG-3’ |
| 23 | BMGF_MIP_Cpar_1 | 5’-GACACGAGAGACTGTAGAGTCGACTTCAGCTTCCCGATATCCGACGGTAGTGTNNNNNNNNNCGCACCGTCTCCACTC-3’ |
| 24 | BMGF_MIP_Cpar_2 | 5’-TCACGTAAGTGGGCATCGTCGACTTCAGCTTCCCGATATCCGACGGTAGTGTNNNNNNNNNCTGACGCGTCGGTATG-3’ |
| 25 | BMGF_MIP_Cpar_3 | 5’-GATAGGCATGGTTTGGTTGGTCGACTTCAGCTTCCCGATATCCGACGGTAGTGTNNNNNNNNNCGTGAAAGGCTCATCGT-3’ |
| 26 | BMGF_MIP_Ctro_1 | 5’-GGTAGGGTGCTTTATGTGTGTGACTTCAGCTTCCCGATATCCGACGGTAGTGTNNNNNNNNNTCTAATTCTTGCTTCTCCTCC-3’ |
| 27 | BMGF_MIP_Ctro_2 | 5’-CATACAAGATTGTCATTACCTGTGACTTCAGCTTCCCGATATCCGACGGTAGTGTNNNNNNNNNGAGCATTAGACGTAGATCTAGC-3’ |
| 28 | BMGF_MIP_Ctro_3 | 5’-GAGGCCACAAACTTATCATTTGTGACTTCAGCTTCCCGATATCCGACGGTAGTGTNNNNNNNNNGTAAAAAATTCCAGAAATGACC-3’ |
| 29 | BMGF_MIP_Ecol_1 | 5’-ACACATAATGCGCATCACCGGACACTTCAGCTTCCCGATATCCGACGGTAGTGTNNNNNNNNNCAGCAAAGTCCATTTCGA-3’ |
| 30 | BMGF_MIP_Ecol_2 | 5’-GTAAGGATAGTAACGACGTTGGACACTTCAGCTTCCCGATATCCGACGGTAGTGTNNNNNNNNNCGATGACGTGGGTCA-3’ |
| 31 | BMGF_MIP_Ecol_3 | 5’-CTGGAACTGGCTGGCTTGGACACTTCAGCTTCCCGATATCCGACGGTAGTGTNNNNNNNNNCGACGCAGAGTGGGA-3’ |
| 32 | BMGF_MIP_Ecol_4 | 5’-TTAACTTTACTCCCTTCCTCGGACACTTCAGCTTCCCGATATCCGACGGTAGTGTNNNNNNNNNTGCGGGTAACGTCAATG-3’ |
| 33 | BMGF_MIP_Efae_1 | 5’-ATTAAGCCAACAAAGCGATCCTGCACTTCAGCTTCCCGATATCCGACGGTAGTGTNNNNNNNNNAATCAAACATTTTATCTACAACGTC-3’ |
| 34 | BMGF_MIP_Efae_2 | 5’-TCTGGCGTGATTAAATCAGCTGCACTTCAGCTTCCCGATATCCGACGGTAGTGTNNNNNNNNNTTGCCACCTACACGTTG-3’ |
| 35 | BMGF_MIP_Efae_3 | 5’-CCGATCTCAACAACTTCTAACTGCACTTCAGCTTCCCGATATCCGACGGTAGTGTNNNNNNNNNGGGAACAGAACCAACACT-3’ |
| 36 | BMGF_MIP_Efae_4 | 5’-TAATAGTCCGTAAGAAGTCCCTGCACTTCAGCTTCCCGATATCCGACGGTAGTGTNNNNNNNNNGAAGTCTCCTGGGATTCC-3’ |
| 37 | BMGF_MIP_Efae_5 | 5’-CCGCCGAATGACTTTAGCTGCACTTCAGCTTCCCGATATCCGACGGTAGTGTNNNNNNNNNCCGATTGGTTTCCAAA-3’ |
| 38 | BMGF_MIP_Efae_6 | 5’-GTTTATGCCGCATGGCATACTGCACTTCAGCTTCCCGATATCCGACGGTAGTGTNNNNNNNNNACTTGGAAACAGGTGCTAATA-3’ |
| 39 | BMGF_MIP_Efae_7 | 5’-TGTTCCGGATCTGTAAATTCCTGCACTTCAGCTTCCCGATATCCGACGGTAGTGTNNNNNNNNNCCAATTCATCCGAATCTC-3’ |
| 40 | BMGF_MIP_Efae_8 | 5’-TACGAAGAAAATGGGAACCACTGCACTTCAGCTTCCCGATATCCGACGGTAGTGTNNNNNNNNNTCTTGCCAGTTGGGATTA-3’ |
| 41 | BMGF_MIP_Efae_9 | 5’-GCACACTAAACGGTAAATTCCTGCACTTCAGCTTCCCGATATCCGACGGTAGTGTNNNNNNNNNTCGATAGGTATCTTCTAATGCC-3’ |
| 42 | BMGF_MIP_Efae_10 | 5’-ACTACGAGTGCCATTAGTTTCTGCACTTCAGCTTCCCGATATCCGACGGTAGTGTNNNNNNNNNCAACCTACCGAGTTGAAAAA-3’ |
| 43 | BMGF_MIP_Efae_11 | 5’-TTGAAAAATGCGGTCGGTACTGCACTTCAGCTTCCCGATATCCGACGGTAGTGTNNNNNNNNNCGAGTTTCTCAAATTACAACC-3’ |
| 44 | BMGF_MIP_Efae_12 | 5’-ACAAATCCGGTAACTGATAGCTGCACTTCAGCTTCCCGATATCCGACGGTAGTGTNNNNNNNNNCCTTCTCCACAAAAGTATTCTGT-3’ |
| 45 | BMGF_MIP_Efam_1 | 5’-CGGTAAAGGGAAATTACAAGTACCACTTCAGCTTCCCGATATCCGACGGTAGTGTNNNNNNNNNCACAAGTGGATCGGC-3’ |
| 46 | BMGF_MIP_Efam_2 | 5’-GTATAAACGGCGGAAGCATACCACTTCAGCTTCCCGATATCCGACGGTAGTGTNNNNNNNNNGTAAAGAACACTTTTGTCTCACG-3’ |
| 47 | BMGF_MIP_Efam_3 | 5’-CATTCCCCGTTCGTTTTGTACCACTTCAGCTTCCCGATATCCGACGGTAGTGTNNNNNNNNNAGCCACTCTTGTGCTGTC-3’ |
| 48 | BMGF_MIP_Efam_4 | 5’-GCGAAAATGTCTACAGTATTTACCACTTCAGCTTCCCGATATCCGACGGTAGTGTNNNNNNNNNACATGGAACCAAAAGAAGTC-3’ |
| 49 | BMGF_MIP_Efam_5 | 5’-CAGTTACGCATTGATCTCATTACCACTTCAGCTTCCCGATATCCGACGGTAGTGTNNNNNNNNNCCTCGCTGGGGCATGTTG-3’ |
| 50 | BMGF_MIP_Efam_6 | 5’-GTTACGCATTGATCTCATTTATACCACTTCAGCTTCCCGATATCCGACGGTAGTGTNNNNNNNNNCTGGGGCATGTTGCA-3’ |
| 51 | BMGF_MIP_Efam_7 | 5’-CGTATAAAACAACCGCATCTTACCACTTCAGCTTCCCGATATCCGACGGTAGTGTNNNNNNNNNTCTTCTACTTTCCCGATTTCT-3’ |
| 52 | BMGF_MIP_Efam_8 | 5’-CAGAAGCGGTCCAAAAAGTACCACTTCAGCTTCCCGATATCCGACGGTAGTGTNNNNNNNNNTCAAAAAAAAGATTGGGGAC-3’ |
| 53 | BMGF_MIP_Hinf_1 | 5’-GAGTGAAGCGAAAGGTGTCGAAGCTTCAGCTTCCCGATATCCGACGGTAGTGTNNNNNNNNNCCACTCAGACCCACCA-3’ |
| 54 | BMGF_MIP_Hinf_2 | 5’-ACCATATTGTGGTACTAAGCCGAAGCTTCAGCTTCCCGATATCCGACGGTAGTGTNNNNNNNNNTCAAAGAGGGTGATAATCCC-3’ |
| 55 | BMGF_MIP_Hinf_3 | 5’-AGAGTGAGTGAAACGAAAGCGAAGCTTCAGCTTCCCGATATCCGACGGTAGTGTNNNNNNNNNGGTTCAAGTCCACTCAGACC-3’ |
| 56 | BMGF_MIP_Koxy_1 | 5’-CATCTATGCACTTCTGCCTCGAGCTTCAGCTTCCCGATATCCGACGGTAGTGTNNNNNNNNNCATAATCATGCGCCGTA-3’ |
| 57 | BMGF_MIP_Kpne_1 | 5’-CAGCGTGCCTTCTCCCGTTAGCTTCAGCTTCCCGATATCCGACGGTAGTGTNNNNNNNNNCATCCGCAGGGACTTC-3’ |
| 58 | BMGF_MIP_Kpne_2 | 5’-AAAAACGGGTTGACTCTGAGTTAGCTTCAGCTTCCCGATATCCGACGGTAGTGTNNNNNNNNNCGGTTCGGTTGCAGAG-3’ |
| 59 | BMGF_MIP_Kpne_3 | 5’-GCCGTGTTTTGCACGGAGTTAGCTTCAGCTTCCCGATATCCGACGGTAGTGTNNNNNNNNNCAGCCCGCATGGATG-3’ |
| 60 | BMGF_MIP_Kpne_4 | 5’-TTCGGGTTATGGTTCAGGGTTAGCTTCAGCTTCCCGATATCCGACGGTAGTGTNNNNNNNNNGTCACGGTCAGCCCA-3’ |
| 61 | BMGF_MIP_Kpne_5 | 5’-AGGTGAAGTCCCTGCGGTTAGCTTCAGCTTCCCGATATCCGACGGTAGTGTNNNNNNNNNCTTCGGGAGAAGGCAC-3’ |
| 62 | BMGF_MIP_Kpne_6 | 5’-CTCGGCCGGGTAAAGTGTTAGCTTCAGCTTCCCGATATCCGACGGTAGTGTNNNNNNNNNGCATTGGCGTTAGGTTT-3’ |
| 63 | BMGF_MIP_Kpne_7 | 5’-GTAGGTGAAGTCCCTGCGTTAGCTTCAGCTTCCCGATATCCGACGGTAGTGTNNNNNNNNNGTAACTTCGGGAGAAGGC-3’ |
| 64 | BMGF_MIP_Kpne_8 | 5’-TCCAGGTGGCCAGCCGTTAGCTTCAGCTTCCCGATATCCGACGGTAGTGTNNNNNNNNNACGCTCTGGTTCCGG-3’ |
| 65 | BMGF_MIP_*mecA*_1 | 5’-CAATCGTTGACGATAATAGCATAGGCTTCAGCTTCCCGATATCCGACGGTAGTGTNNNNNNNNNCTCCAACATGAAGATGGC-3’ |
| 66 | BMGF_MIP_*mecA*_2 | 5’-TCCGTAACGATGGTTGCTATAGGCTTCAGCTTCCCGATATCCGACGGTAGTGTNNNNNNNNNATACGATATAGATGAATAACAAAAC-3’ |
| 67 | BMGF_MIP_*mecA*_3 | 5’-TGTGGAATTGGCCAATACAATAGGCTTCAGCTTCCCGATATCCGACGGTAGTGTNNNNNNNNNAAATCAGAACGTGGTAAAATTTTAG-3’ |
| 68 | BMGF_MIP_*mecA*_4 | 5’-CCTTGCTCAAATTTTTTGGCAAGCGCTTCAGCTTCCCGATATCCGACGGTAGTGTNNNNNNNNNTTTTCACCGATTCCCAA-3’ |
| 69 | BMGF_MIP_*mecA*_5 | 5’-CGGGATATTTTCACCGATTAAGCGCTTCAGCTTCCCGATATCCGACGGTAGTGTNNNNNNNNNTGAGATTTGTGCTTTATAAAAGG-3’ |
| 70 | BMGF_MIP_*mecA*_6 | 5’-AATATCCCGAGTGATTATCCAAGCGCTTCAGCTTCCCGATATCCGACGGTAGTGTNNNNNNNNNAAGGTATGCAAGATTTGGG-3’ |
| 71 | BMGF_MIP_Pmirab_1 | 5’-CCAGACAACCCCGACCGAATGCTTCAGCTTCCCGATATCCGACGGTAGTGTNNNNNNNNNTAAACACTATATGTTGCGGTTAAC-3’ |
| 72 | BMGF_MIP_Nmen_1 | 5’-CAAGACAAAAGCAACGGCTGCTGCTTCAGCTTCCCGATATCCGACGGTAGTGTNNNNNNNNNCACAGCACCATTTACCG-3’ |
| 73 | BMGF_MIP_Nmen_2 | 5’-GTAACCGTAGCAACTGAACTGCTGCTTCAGCTTCCCGATATCCGACGGTAGTGTNNNNNNNNNCGAAATTCCAACATAACCG-3’ |
| 74 | BMGF_MIP_Nmen_3 | 5’-CTATACCGTTGTGGTGGATGCTGCTTCAGCTTCCCGATATCCGACGGTAGTGTNNNNNNNNNGGCATTCTGAAACGCA-3’ |
| 75 | BMGF_MIP_Paer_1 | 5’-AGCATGGGAGACACACGACTTGCTTCAGCTTCCCGATATCCGACGGTAGTGTNNNNNNNNNTGCAAACTCCGAATACCC-3’ |
| 76 | BMGF_MIP_Paer_2 | 5’-GACGAAGAGTAAATGCTTGAACTTGCTTCAGCTTCCCGATATCCGACGGTAGTGTNNNNNNNNNCCTATAATGCGCACCAC-3’ |
| 77 | BMGF_MIP_Paer_3 | 5’-CGTCACGTGCCGCACACTTGCTTCAGCTTCCCGATATCCGACGGTAGTGTNNNNNNNNNGCAGTGATATCGAAGAGGG-3’ |
| 78 | BMGF_MIP_Paer_4 | 5’-GACGAAGATCTGTTCCTGACTTGCTTCAGCTTCCCGATATCCGACGGTAGTGTNNNNNNNNNATGTTCTCGTCCCAGTCGC-3’ |
| 79 | BMGF_MIP_Paer_5 | 5’-ACCTACCAGGAAGGCCAACTTGCTTCAGCTTCCCGATATCCGACGGTAGTGTNNNNNNNNNGCCTTCACCGAGGATTC-3’ |
| 80 | BMGF_MIP_Paer_6 | 5’-GTCCATCACCGGCACCACTTGCTTCAGCTTCCCGATATCCGACGGTAGTGTNNNNNNNNNGTATCCATGGCAACCC-3’ |
| 81 | BMGF_MIP_Paer_7 | 5’-GCCGTGATTCCCTGGTTACTTGCTTCAGCTTCCCGATATCCGACGGTAGTGTNNNNNNNNNCGTCCATGTGATCGTCTT-3’ |
| 82 | BMGF_MIP_Paer_8 | 5’-CCTGTCGTTCCTCATCGACTTGCTTCAGCTTCCCGATATCCGACGGTAGTGTNNNNNNNNNAAACACCCCTCGCTGA-3’ |
| 83 | BMGF_MIP_Paer_9 | 5’-GGCATTGACCGATGCCAACTTGCTTCAGCTTCCCGATATCCGACGGTAGTGTNNNNNNNNNCCAATGGCAGAAGGAAG-3’ |
| 84 | BMGF_MIP_Saga_1 | 5’-ACACCTTTGTTTCCGTGACACAACCTTCAGCTTCCCGATATCCGACGGTAGTGTNNNNNNNNNGCATATCTTCAACAGGAACAAT-3’ |
| 85 | BMGF_MIP_Saga_2 | 5’-CGTCAAGTGCAACTCTTACACAACCTTCAGCTTCCCGATATCCGACGGTAGTGTNNNNNNNNNTTTGCTTGTTTATTTGATCG-3’ |
| 86 | BMGF_MIP_Saga_3 | 5’-GGACCAATATGATCGGGACAACCTTCAGCTTCCCGATATCCGACGGTAGTGTNNNNNNNNNGAAAAAATAATCTTCGAATGTAGG-3’ |
| 87 | BMGF_MIP_Saur_1 | 5’-CTTAGGTACTGTCCCTAATGCACACCTTCAGCTTCCCGATATCCGACGGTAGTGTNNNNNNNNNCTTGTGCAAGTAAGACACGAG-3’ |
| 88 | BMGF_MIP_Saur_2 | 5’-CGTATAACGCTAATCTTTTAAAACACACCTTCAGCTTCCCGATATCCGACGGTAGTGTNNNNNNNNNGGCTTGTTATATTAACAGATCATG-3’ |
| 89 | BMGF_MIP_Saur_3 | 5’-ACTACCATCGACGCTAAGCACACCTTCAGCTTCCCGATATCCGACGGTAGTGTNNNNNNNNNAACGTTCTACTCTAGCGGAA-3’ |
| 90 | BMGF_MIP_Saur_4 | 5’-TAGAGCATAGAACATTGATTTATCACACCTTCAGCTTCCCGATATCCGACGGTAGTGTNNNNNNNNNGTTCAGTCAACTACTGCCAA-3’ |
| 91 | BMGF_MIP_Saur_5 | 5’-TCGACTACAGGATTATTACCCACACCTTCAGCTTCCCGATATCCGACGGTAGTGTNNNNNNNNNCTCAGGATCCACTCAAGAGA-3’ |
| 92 | BMGF_MIP_Saur_6 | 5’-CACGCTTCGCCTATCCTCACACCTTCAGCTTCCCGATATCCGACGGTAGTGTNNNNNNNNNCCTTACTGCTTAGACGTGC-3’ |
| 93 | BMGF_MIP_Sepi_1 | 5’-CAGAATTAATGTTAAACCGTACTAGGCCTTCAGCTTCCCGATATCCGACGGTAGTGTNNNNNNNNNCGTGCTGACTTACGAGTTAAC-3’ |
| 94 | BMGF_MIP_Sepi_2 | 5’-TTGTGCTACACGACCGATTAGGCCTTCAGCTTCCCGATATCCGACGGTAGTGTNNNNNNNNNCGTTGTCCATAATTACTTTATCGT-3’ |
| 95 | BMGF_MIP_Shae_1 | 5’-GATAACCCACTCACTTAAGTCTTGCCTTCAGCTTCCCGATATCCGACGGTAGTGTNNNNNNNNNGTTCTAAAATAGTTCTCTAAAGTCG-3’ |
| 96 | BMGF_MIP_Shae_2 | 5’-ACGGATTAGCCATCGAACCTTGCCTTCAGCTTCCCGATATCCGACGGTAGTGTNNNNNNNNNTAAAGCAGTAGGCAATGCC-3’ |
| 97 | BMGF_MIP_Shae_3 | 5’-AAGAGACGAGGATACTTGTACTTGCCTTCAGCTTCCCGATATCCGACGGTAGTGTNNNNNNNNNGTCTGATCGATGGCCTTGTG-3’ |
| 98 | BMGF_MIP_Slug_1 | 5’-TCAAAGTGCAATACTCAAGCTTACCCTTCAGCTTCCCGATATCCGACGGTAGTGTNNNNNNNNNCGACGACCTACCAGAAGGT-3’ |
| 99 | BMGF_MIP_Slug_2 | 5’-ACGCTGATCACACTGACATTACCCTTCAGCTTCCCGATATCCGACGGTAGTGTNNNNNNNNNCCGACAGTGGAAACGAAT-3’ |
| 100 | BMGF_MIP_Slug_3 | 5’-GTATAGCAGTGGTAATACGTTTACCCTTCAGCTTCCCGATATCCGACGGTAGTGTNNNNNNNNNCCAGTACAACGCCGTATTT-3’ |
| 101 | BMGF_MIP_Smut_1 | 5’-GGGTTCGTTTTAAAGCATGTGATCCCTTCAGCTTCCCGATATCCGACGGTAGTGTNNNNNNNNNTCACCTGACTAATAGCAGCG-3’ |
| 102 | BMGF_MIP_Smut_2 | 5’-TAAGTGTCCGCATGACTTCGATCCCTTCAGCTTCCCGATATCCGACGGTAGTGTNNNNNNNNNTTTCTTCTATGGCGGTAACA-3’ |
| 103 | BMGF_MIP_Spne_1 | 5’-GTTTACGAATCTCTTTCCACAGGTCCTTCAGCTTCCCGATATCCGACGGTAGTGTNNNNNNNNNAAAGTTCGAAAGGCTTTATTC-3’ |
| 104 | BMGF_MIP_Spne_2 | 5’-ACAAACCAAGTACTAAGAGCAGGTCCTTCAGCTTCCCGATATCCGACGGTAGTGTNNNNNNNNNATCTTTTTGAATCTTATCCGC-3’ |
| 105 | BMGF_MIP_Spne_3 | 5’-GACCAGGAGTATAACCCTAGGTCCTTCAGCTTCCCGATATCCGACGGTAGTGTNNNNNNNNNGCAATCTTCTCTTGACTGACG-3’ |
| 106 | BMGF_MIP_Spne_4 | 5’-AGAAGCAAGTGACAAAGACAGGTCCTTCAGCTTCCCGATATCCGACGGTAGTGTNNNNNNNNNTGGCTGTGATATACTAATATAGTT-3’ |
| 107 | BMGF_MIP_Spne_5 | 5’-TGCCCGAATTAACTTAAAAGTTAGGTCCTTCAGCTTCCCGATATCCGACGGTAGTGTNNNNNNNNNGAAAAGTACTTCCAACCAGAAC-3’ |
| 108 | BMGF_MIP_Spyo_1 | 5’-TTGTCAGTAATGCCCCGAGCCATCTTCAGCTTCCCGATATCCGACGGTAGTGTNNNNNNNNNCTCCAAGCCAGGTAACAATA-3’ |
| 109 | BMGF_MIP_Spyo_2 | 5’-CGTATATGGCTATACACTAGGCCATCTTCAGCTTCCCGATATCCGACGGTAGTGTNNNNNNNNNCTTTTCAAATGGAACAGCAG-3’ |
| 110 | BMGF_MIP_Spyo_3 | 5’-TCCTATATCTCTGAAAAAAGCGCCATCTTCAGCTTCCCGATATCCGACGGTAGTGTNNNNNNNNNTAGAAATGTTGGCATCGC-3’ |
| 111 | BMGF_MIP_Ssal_1 | 5’-ATCCAACACCTAGCACTCTCAGTCTTCAGCTTCCCGATATCCGACGGTAGTGTNNNNNNNNNTAGCTGCGGCACTGAAT-3’ |
| 112 | BMGF_MIP_Ssal_2 | 5’-CGTCCCGAGGTCATAGTCAGTCTTCAGCTTCCCGATATCCGACGGTAGTGTNNNNNNNNNCCCAACAACGCCTCAC-3’ |
| 113 | BMGF_MIP_Ssal_3 | 5’-TATGGTCAGCCGCAGTGTCAGTCTTCAGCTTCCCGATATCCGACGGTAGTGTNNNNNNNNNCGTAACTTCGGGAGAAGG-3’ |
| 114 | BMGF_MIP_Ssang_1 | 5’-GGAACGGTTCTTGGGACAACGTCTTCAGCTTCCCGATATCCGACGGTAGTGTNNNNNNNNNGAAGACCTGGATAGGCTTAAAG-3’ |
| 115 | BMGF_MIP_Ssang_2 | 5’-ATCGTTGATCAGATGGAACAACGTCTTCAGCTTCCCGATATCCGACGGTAGTGTNNNNNNNNNGATTGACAATCAGACTTACACTAG-3’ |
| 116 | BMGF_MIP_Ssapr_1 | 5’-ATAGCCGACAGTCCTAGATGGCTCTTCAGCTTCCCGATATCCGACGGTAGTGTNNNNNNNNNTCCGGGCAAGATGACGAG-3’ |
| 117 | BMGF_MIP_Ssapr_2 | 5’-ACTTATAATGCGATATTTGATGGCTCTTCAGCTTCCCGATATCCGACGGTAGTGTNNNNNNNNNATATTTATATTTCGGATTTATTACG-3’ |
| 118 | BMGF_MIP_Staph_1 | 5’-TAAGGTTTCCAGAGGAAGGCAGTTCTTCAGCTTCCCGATATCCGACGGTAGTGTNNNNNNNNNGGTGAGAATCCCGTCC-3’ |
| 119 | BMGF_MIP_Staph_2 | 5’-ACGGAGCACGTGAAATTCCAGTTCTTCAGCTTCCCGATATCCGACGGTAGTGTNNNNNNNNNAATGTTGTCTCTCTTGAGTGGAT-3’ |
| 120 | BMGF_MIP_Staph_3 | 5’-ACTGAGGGTAGCGGAGACAGTTCTTCAGCTTCCCGATATCCGACGGTAGTGTNNNNNNNNNAACCGACTTACGTTGAAAAGT-3’ |
| 121 | BMGF_MIP_Strep_1 | 5’-ACTTGTTGTAGGCACACGGTGCACTTCAGCTTCCCGATATCCGACGGTAGTGTNNNNNNNNNCACGCTCTCACCCATTA-3’ |
| 122 | BMGF_MIP_Strep_2 | 5’-AGGTCCCGACTAACCCAGTGCACTTCAGCTTCCCGATATCCGACGGTAGTGTNNNNNNNNNCATCGGATACACCTTTCG-3’ |
| 123 | BMGF_MIP_Strep_3 | 5’-CGACCCTCTGATTACAAATGTGCACTTCAGCTTCCCGATATCCGACGGTAGTGTNNNNNNNNNAATGCCGGCTACATGA-3’ |
| 124 | BMGF_MIP_Strep_4 | 5’-ACGTATTACCGCGGCTGGTGCACTTCAGCTTCCCGATATCCGACGGTAGTGTNNNNNNNNNCAATAAATCCGGACAACG-3’ |
| 125 | BMGF_MIP_Strep_5 | 5’-ATAACCGCTACACTACGGGTGCACTTCAGCTTCCCGATATCCGACGGTAGTGTNNNNNNNNNGATCTTCGCCGTGACA-3’ |
| 126 | BMGF_MIP_Strep_6 | 5’-CTTAGTCTTACGGTGGACGTGCACTTCAGCTTCCCGATATCCGACGGTAGTGTNNNNNNNNNGGCGGACGAGCCTTC-3’ |
| 127 | BMGF_MIP_Strep_7 | 5’-AGCACTCGCAGTCTGACGTGCACTTCAGCTTCCCGATATCCGACGGTAGTGTNNNNNNNNNGCTGTTTCCCTTTCGAC-3’ |
| 128 | BMGF_MIP_vanA_1 | 5’-CGTCCTCGCTCCTCTGAGCACCTTCAGCTTCCCGATATCCGACGGTAGTGTNNNNNNNNNGCTTTATATATTTTTTTTGCCGT-3’ |
| 129 | BMGF_MIP_vanA_2 | 5’-GGAAGTCGAGCCGGAAAAGCACCTTCAGCTTCCCGATATCCGACGGTAGTGTNNNNNNNNNCTGCAGTACGGAATCTTTC-3’ |
| 130 | BMGF_MIP_vanA_3 | 5’-AACGCGGCACTGTTTCCAGCACCTTCAGCTTCCCGATATCCGACGGTAGTGTNNNNNNNNNCCTGATTTGGTCCACCTC-3’ |
| 131 | BMGF_MIP_EC1 | 5’-AAGAGATGCTCGACTGGGACACTTCAGCTTCCCGATATCCGACGGTAGTGTNNNNNNNNNCGTACCTCGCATTAC-3’ |
| 132 | BMGF_MIP_Koxy2 | 5’-GGCGCTGCAGCAGGTCGAGCTTCAGCTTCCCGATATCCGACGGTAGTGTNNNNNNNNNGTGACGGCACCACGC-3’ |
| 133 | BMGF_MIP_Kpneu2 | 5’-ACGGCTTCTTGTGAATGTTAGCTTCAGCTTCCCGATATCCGACGGTAGTGTNNNNNNNNNTACCTTTGACCCAGG-3’ |
| 134 | BMGF_MIP_PA3 | 5’-GGTGGTCGACAGGTCGACTTGCTTCAGCTTCCCGATATCCGACGGTAGTGTNNNNNNNNNACTGGCTGGAAATCG-3’ |
| 135 | BMGF_MIP_PA5 | 5’-GAACATCAAGGTGTTCACTTGCTTCAGCTTCCCGATATCCGACGGTAGTGTNNNNNNNNNTGCCGATCGGCCACG-3’ |
| 136 | BMGF_MIP_PA6 | 5’-AGGGAGAAAGTGAGAACTTGCTTCAGCTTCCCGATATCCGACGGTAGTGTNNNNNNNNNGGCGCTAATACCGC-3’ |
| 137 | BMGF_MIP_Pmira1 | 5’-GCGGTGGTTGCCCCGAATGCTTCAGCTTCCCGATATCCGACGGTAGTGTNNNNNNNNNTGCGCATTCTCAAAC-3’ |
| 138 | BMGF_MIP_Pmira2 | 5’-CATTACGTCGTCGTTGAATGCTTCAGCTTCCCGATATCCGACGGTAGTGTNNNNNNNNNTTTGCTAAAGGTAGC-3’ |
| 139 | BMGF_MIP_SA2 | 5’-TGCTTTGTTTCAGGTCACACCTTCAGCTTCCCGATATCCGACGGTAGTGTNNNNNNNNNCCATATTTCTCTACACC-3’ |
| 140 | BMGF_MIP_mecA1 | 5’-AGTGCTAATAATTCAATAGGCTTCAGCTTCCCGATATCCGACGGTAGTGTNNNNNNNNNATAGACGTCATATGAAGG-3’ |
| 141 | BMGF_MIP_KPC | 5’-CGGCGTTATCACTGTAATGCCTTCAGCTTCCCGATATCCGACGGTAGTGTNNNNNNNNNCCAACTCCTTCAGCAAC-3’ |
| 142 | BMGF_MIP_NDM2 | 5’-TGCCAGACATTCGGTGCGATCTTCAGCTTCCCGATATCCGACGGTAGTGTNNNNNNNNNCCGGCATGTCGAGATA-3’ |
| 143 | BMGF_MIP_Oxa-48 | 5’-ATTATTGGTAAATCCTCTACCTTCAGCTTCCCGATATCCGACGGTAGTGTNNNNNNNNNTAAAAATGCTTGGTTCG-3’ |
| 144 | BMGF_MIP_VIM | 5’-AAAGCAACTCATCACCATGGACTTCAGCTTCCCGATATCCGACGGTAGTGTNNNNNNNNNCGCACCCCACGCTG-3’ |
