# Supplementary material for: Highly multiplexed targeted sequencing strategy for infectious disease surveillance
Source: BMC Biotechnol. 2023 Aug 23;23:31. doi: 10.1186/s12896-023-00804-7 (PMC10463907; doi:10.1186/s12896-023-00804-7)
Supplement: Supplementary file 3 — Supplementary Material 3 [file 12896_2023_804_MOESM3_ESM.docx]

**Supplementary table 3:** Truseq HT DNA combinatorial dual (CD) index adapters (Illumina) as used for sample multiplexing.

| Sample ID | Index 1 (i7) adapters | 8 nt | Index 2 (i5) adapters | 8 nt |
| --- | --- | --- | --- | --- |
| 1 | D701 | 5’-ATTACTCG-3’ | D501 | 5’-TATAGCCT-3’ |
| 2 | D702 | 5’-TCCGGAGA-3’ | D501 | 5’-TATAGCCT-3’ |
| 3 | D703 | 5’-CGCTCATT-3’ | D501 | 5’-TATAGCCT-3’ |
| 4 | D704 | 5’-GAGATTCC-3’ | D501 | 5’-TATAGCCT-3’ |
| 5 | D705 | 5’-ATTCAGAA-3’ | D501 | 5’-TATAGCCT-3’ |
| 6 | D706 | 5’-GAATTCGT-3’ | D501 | 5’-TATAGCCT-3’ |
| 7 | D707 | 5’-CTGAAGCT-3’ | D501 | 5’-TATAGCCT-3’ |
| 8 | D708 | 5’-TAATGCGC-3’ | D501 | 5’-TATAGCCT-3’ |
| 9 | D709 | 5’-CGGCTATG-3’ | D501 | 5’-TATAGCCT-3’ |
| 10 | D710 | 5’-TCCGCGAA-3’ | D501 | 5’-TATAGCCT-3’ |
| 11 | D711 | 5’-TCTCGCGC-3’ | D501 | 5’-TATAGCCT-3’ |
| 12 | D712 | 5’-AGCGATAG-3’ | D501 | 5’-TATAGCCT-3’ |
| 13 | D701 | 5’-ATTACTCG-3’ | D502 | 5’-ATAGAGGC-3’ |
| 14 | D702 | 5’-TCCGGAGA-3’ | D502 | 5’-ATAGAGGC-3’ |
| 15 | D703 | 5’-CGCTCATT-3’ | D502 | 5’-ATAGAGGC-3’ |
| 16 | D704 | 5’-GAGATTCC-3’ | D502 | 5’-ATAGAGGC-3’ |
| 17 | D705 | 5’-ATTCAGAA-3’ | D502 | 5’-ATAGAGGC-3’ |
| 18 | D706 | 5’-GAATTCGT-3’ | D502 | 5’-ATAGAGGC-3’ |
| 19 | D707 | 5’-CTGAAGCT-3’ | D502 | 5’-ATAGAGGC-3’ |
| 20 | D708 | 5’-TAATGCGC-3’ | D502 | 5’-ATAGAGGC-3’ |
| 21 | D709 | 5’-CGGCTATG-3’ | D502 | 5’-ATAGAGGC-3’ |
| 22 | D710 | 5’-TCCGCGAA-3’ | D502 | 5’-ATAGAGGC-3’ |
| 23 | D711 | 5’-TCTCGCGC-3’ | D502 | 5’-ATAGAGGC-3’ |
| 24 | D712 | 5’-AGCGATAG-3’ | D502 | 5’-ATAGAGGC-3’ |
| 25 | D701 | 5’-ATTACTCG-3’ | D503 | 5’-CCTATCCT-3’ |
| 26 | D702 | 5’-TCCGGAGA-3’ | D503 | 5’-CCTATCCT-3’ |
| 27 | D703 | 5’-CGCTCATT-3’ | D503 | 5’-CCTATCCT-3’ |
| 28 | D704 | 5’-GAGATTCC-3’ | D503 | 5’-CCTATCCT-3’ |
| 29 | D705 | 5’-ATTCAGAA-3’ | D503 | 5’-CCTATCCT-3’ |
| 30 | D706 | 5’-GAATTCGT-3’ | D503 | 5’-CCTATCCT-3’ |
| 31 | D707 | 5’-CTGAAGCT-3’ | D503 | 5’-CCTATCCT-3’ |
| 32 | D708 | 5’-TAATGCGC-3’ | D503 | 5’-CCTATCCT-3’ |
| 33 | D709 | 5’-CGGCTATG | D503 | 5’-CCTATCCT-3’ |
| 34 | D710 | 5’-TCCGCGAA | D503 | 5’-CCTATCCT-3’ |
| 35 | D711 | 5’-TCTCGCGC-3’ | D503 | 5’-CCTATCCT-3’ |
| 36 | D712 | 5’-AGCGATAG-3’ | D503 | 5’-CCTATCCT-3’ |
| 37 | D701 | 5’-ATTACTCG-3’ | D504 | 5’-GGCTCTGA-3’ |
| 38 | D702 | 5’-TCCGGAGA | D504 | 5’-GGCTCTGA-3’ |
| 39 | D703 | 5’-CGCTCATT-3’ | D504 | 5’-GGCTCTGA-3’ |
| 40 | D704 | 5’-GAGATTCC-3’ | D504 | 5’-GGCTCTGA-3’ |
| 41 | D705 | 5’-ATTCAGAA-3’ | D504 | 5’-GGCTCTGA-3’ |
| 42 | D706 | 5’-GAATTCGT-3’ | D504 | 5’-GGCTCTGA-3’ |
| 43 | D707 | 5’-CTGAAGCT-3’ | D504 | 5’-GGCTCTGA-3’ |
| 44 | D708 | 5’-TAATGCGC-3’ | D504 | 5’-GGCTCTGA-3’ |
| 45 | D709 | 5’-CGGCTATG-3’ | D504 | 5’-GGCTCTGA-3’ |
| 46 | D710 | 5’-TCCGCGAA-3’ | D504 | 5’-GGCTCTGA-3’ |
| 47 | D711 | 5’-TCTCGCGC-3’ | D504 | 5’-GGCTCTGA-3’ |
| 48 | D712 | 5’-AGCGATAG-3’ | D504 | 5’-GGCTCTGA-3’ |
| 49 | D701 | 5’-ATTACTCG-3’ | D505 | 5’-AGGCGAAG-3’ |
| 50 | D702 | 5’-TCCGGAGA-3’ | D505 | 5’-AGGCGAAG-3’ |
| 51 | D703 | 5’-CGCTCATT-3’ | D505 | 5’-AGGCGAAG-3’ |
| 52 | D704 | 5’-GAGATTCC-3’ | D505 | 5’-AGGCGAAG-3’ |
| 53 | D705 | 5’-ATTCAGAA-3’ | D505 | 5’-AGGCGAAG-3’ |
| 54 | D706 | 5’-GAATTCGT-3’ | D505 | 5’-AGGCGAAG-3’ |
| 55 | D707 | 5’-CTGAAGCT-3’ | D505 | 5’-AGGCGAAG-3’ |
| 56 | D708 | 5’-TAATGCGC-3’ | D505 | 5’-AGGCGAAG-3’ |
| 57 | D709 | 5’-CGGCTATG-3’ | D505 | 5’-AGGCGAAG-3’ |
| 58 | D710 | 5’-TCCGCGAA-3’ | D505 | 5’-AGGCGAAG-3’ |
| 59 | D711 | 5’-TCTCGCGC-3’ | D505 | 5’-AGGCGAAG-3’ |
| 60 | D712 | 5’-AGCGATAG-3’ | D505 | 5’-AGGCGAAG-3’ |
| 61 | D701 | 5’-ATTACTCG-3’ | D506 | 5’-TAATCTTA-3’ |
| 62 | D702 | 5’-TCCGGAGA-3’ | D506 | 5’-TAATCTTA-3’ |
| 63 | D703 | 5’-CGCTCATT-3’ | D506 | 5’-TAATCTTA-3’ |
| 64 | D704 | 5’-GAGATTCC-3’ | D506 | 5’-TAATCTTA-3’ |
| 65 | D705 | 5’-ATTCAGAA-3’ | D506 | 5’-TAATCTTA-3’ |
| 66 | D706 | 5’-GAATTCGT-3’ | D506 | 5’-TAATCTTA-3’ |
| 67 | D707 | 5’-CTGAAGCT-3’ | D506 | 5’-TAATCTTA-3’ |
| 68 | D708 | 5’-TAATGCGC-3’ | D506 | 5’-TAATCTTA-3’ |
| 69 | D709 | 5’-CGGCTATG-3’ | D506 | 5’-TAATCTTA-3’ |
| 70 | D710 | 5’-TCCGCGAA-3’ | D506 | 5’-TAATCTTA-3’ |
| 71 | D711 | 5’-TCTCGCGC-3’ | D506 | 5’-TAATCTTA-3’ |
| 72 | D712 | 5’-AGCGATAG-3’ | D506 | 5’-TAATCTTA-3’ |
| 73 | D701 | 5’-ATTACTCG-3’ | D507 | 5’-CAGGACGT-3’ |
| 74 | D702 | 5’-TCCGGAGA-3’ | D507 | 5’-CAGGACGT-3’ |
| 75 | D703 | 5’-CGCTCATT-3’ | D507 | 5’-CAGGACGT-3’ |
| 76 | D704 | 5’-GAGATTCC-3’ | D507 | 5’-CAGGACGT-3’ |
| 77 | D705 | 5’-ATTCAGAA-3’ | D507 | 5’-CAGGACGT-3’ |
| 78 | D706 | 5’-GAATTCGT-3’ | D507 | 5’-CAGGACGT-3’ |
| 79 | D707 | 5’-CTGAAGCT-3’ | D507 | 5’-CAGGACGT-3’ |
| 80 | D708 | 5’-TAATGCGC-3’ | D507 | 5’-CAGGACGT-3’ |
| 81 | D709 | 5’-CGGCTATG-3’ | D507 | 5’-CAGGACGT-3’ |
| 82 | D710 | 5’-TCCGCGAA-3’ | D507 | 5’-CAGGACGT-3’ |
| 83 | D711 | 5’-TCTCGCGC-3’ | D507 | 5’-CAGGACGT-3’ |
| 84 | D712 | 5’-AGCGATAG-3’ | D507 | 5’-CAGGACGT-3’ |
| 85 | D701 | 5’-ATTACTCG-3’ | D508 | 5’-GTACTGAC-3’ |
| 86 | D702 | 5’-TCCGGAGA-3’ | D508 | 5’-GTACTGAC-3’ |
| 87 | D703 | 5’-CGCTCATT-3’ | D508 | 5’-GTACTGAC-3’ |
| 88 | D704 | 5’-GAGATTCC-3’ | D508 | 5’-GTACTGAC-3’ |
| 89 | D705 | 5’-ATTCAGAA-3’ | D508 | 5’-GTACTGAC-3’ |
| 90 | D706 | 5’-GAATTCGT-3’ | D508 | 5’-GTACTGAC-3’ |
| 91 | D707 | 5’-CTGAAGCT-3’ | D508 | 5’-GTACTGAC-3’ |
| 92 | D708 | 5’-TAATGCGC-3’ | D508 | 5’-GTACTGAC-3’ |
| 93 | D709 | 5’-CGGCTATG-3’ | D508 | 5’-GTACTGAC-3’ |
| 94 | D710 | 5’-TCCGCGAA-3’ | D508 | 5’-GTACTGAC-3’ |
| 95 | D711 | 5’-TCTCGCGC-3’ | D508 | 5’-GTACTGAC-3’ |
| 96 | D712 | 5’-AGCGATAG-3’ | D508 | 5’-GTACTGAC-3’ |
