# Supplementary material for: Highly multiplexed targeted sequencing strategy for infectious disease surveillance
Source: BMC Biotechnol. 2023 Aug 23;23:31. doi: 10.1186/s12896-023-00804-7 (PMC10463907; doi:10.1186/s12896-023-00804-7)
Supplement: Supplementary file 4 — Supplementary Material 4 [file 12896_2023_804_MOESM4_ESM.docx]

**Supplementary table 4**: List of targeted pathogens and antibiotic resistance genes and the corresponding number of designed probes.

| **Pathogen** | **No of Probes** | **Pathogen** | **No of Probes** |
| --- | --- | --- | --- |
| *Aspergillus fumigatus* | 3 | *Staphylococcus epidermidis* | 2 |
| *Candida albicans* | 11 | *Staphylococcus haemolyticus* | 3 |
| *Candida dubliniensis* | 3 | *Staphylococcus lugdunensis* | 3 |
| *Candida glabrata* | 3 | *Staphylococcus saprophyticus* | 2 |
| *Candida parapsilosis* | 3 | *Streptococcus ssp.* | 7 |
| *Candida tropicalis* | 3 | *Streptococcus agalactiae* | 3 |
| *Citrobacter koseri* | 2 | *Streptococcus mutans* | 2 |
| *Eschericia coli* | 5 | *Streptococcus pneumoniae* | 5 |
| *Enterococcus faecalis* | 12 | *Streptococcus pyogenes* | 3 |
| *Enterococcus faecium* | 8 | *Streptococcus salivarius* | 3 |
| *Haemophilus influenzae* | 3 | *Streptococcus sanguinis* | 2 |
| *Klebsiella oxytoca* | 2 | *mec A (resistance gene)* | 4 |
| *Klebsiella pneumoniae* | 9 | *mec ALGA (resistance gene)* | 3 |
| *Neisseria meningitidis* | 3 | *van A (resistance gene)* | 3 |
| *Proteus mirabilis* | 3 | *NDM* *(resistance gene)* | 1 |
| *Pseudomonas aeruginosa* | 12 | *OXA-48* *(resistance gene)* | 1 |
| *Staphylococci ssp.* | 3 | *KPC* *(resistance gene)* | 1 |
| *Staphylococcus aureus* | 7 | *VIM* *(resistance gene)* | 1 |
