# Supplementary material for: Highly multiplexed targeted sequencing strategy for infectious disease surveillance
Source: BMC Biotechnol. 2023 Aug 23;23:31. doi: 10.1186/s12896-023-00804-7 (PMC10463907; doi:10.1186/s12896-023-00804-7)
Supplement: Supplementary file 5 — Supplementary Material 5 [file 12896_2023_804_MOESM5_ESM.docx]

**Supplementary table 5:** Detailed results on assay performance on 24 characterized positive blood cultures (n=2) (Summary results in **Fig.5**). Numbers correspond to the average UMIs obtained for an experimental repetition with technical duplicates. Green color corresponds to true positives, orange colors to low coverage and grey color to false negatives.

| **Plate Sample ID** | *C.glabrata* | *C.parapsilosis* | *K.pneumoniae* | *E.faecalis* | *P.aeruginosa* | *S.epidermidis* | *mecA* | *Staphylococci ssp.* | *E.coli* | Blood culture based identification |
| --- | --- | --- | --- | --- | --- | --- | --- | --- | --- | --- |
| QL-24 | 51.5 |  |  |  |  |  |  |  |  | *C.glabrata* |
| QL-23 | 212 |  |  |  |  |  |  |  |  | *C.glabrata* |
| QL-22 | 614 |  |  |  |  |  |  |  |  | *C.glabrata* |
| QL-21 |  | 182 |  |  |  |  |  |  |  | *C.parapsilosis* |
| QL-20 |  |  | 127157.5 |  |  |  |  |  |  | *K.pneumoniae* |
| QL-19 |  |  | 164831.5 |  |  |  |  |  |  | *K.pneumoniae* |
| QL-18 |  |  | 66379 |  |  |  |  |  |  | *K.pneumoniae* |
| QL-17 |  |  | 3.5 |  |  |  |  |  |  | *K.pneumoniae* |
| QL-16 |  |  | 3.5 |  |  |  |  |  |  | *K.pneumoniae* |
| QL-15 |  |  |  | 0 |  |  |  |  |  | *E.faecalis* |
| QL-14 |  |  |  | 0 |  |  |  |  |  | *E.faecalis* |
| QL-13 |  |  |  | 0 |  |  |  |  |  | *E.faecalis* |
| QL-12 |  |  |  | 0 |  |  |  |  |  | *E.faecalis* |
| QL-11 |  |  |  | 3 |  |  |  |  |  | *E.faecalis* |
| QL-10 |  |  |  |  | 28 |  |  |  |  | *P.aeruginosa* |
| QL-9 |  |  |  |  | 2271.5 |  |  |  |  | *P.aeruginosa* |
| QL-8 |  |  |  |  | 4186 |  |  |  |  | *P.aeruginosa* |
| QL-7 |  |  |  |  | 8606.5 |  |  |  |  | *P.aeruginosa* |
| QL-6 |  |  |  |  | 11.5 |  |  |  |  | *P.aeruginosa* |
| QL-5 |  |  |  |  |  | 36.5 | 16.5 | 117 |  | *S.epidermidis Staphylococci ssp.mecA* |
| QL-4 |  |  |  |  |  | 7 | 3.5 | 17.5 |  | *S.epidermidis Staphylococci ssp.mecA* |
| QL-3 |  |  |  |  |  | 275 | 2.5 | 2062.5 | 465 | *S.epidermidis Staphylococci ssp.mecA* |
| QL-2 |  |  |  |  |  | 320 |  | 8333 |  | *S.epidermidis Staphylococci ssp.* |
| QL-1 |  |  |  |  |  | 320 |  | 23541 |  | *S.epidermidis Staphylococci ssp.* |
| True positive  Low coverage  False negative | | | | | | | | | | |
