# Supplementary figures and images for: Highly multiplexed targeted sequencing strategy for infectious disease surveillance
Source: BMC Biotechnol. 2023 Aug 23;23:31. doi: 10.1186/s12896-023-00804-7 (PMC10463907; doi:10.1186/s12896-023-00804-7)

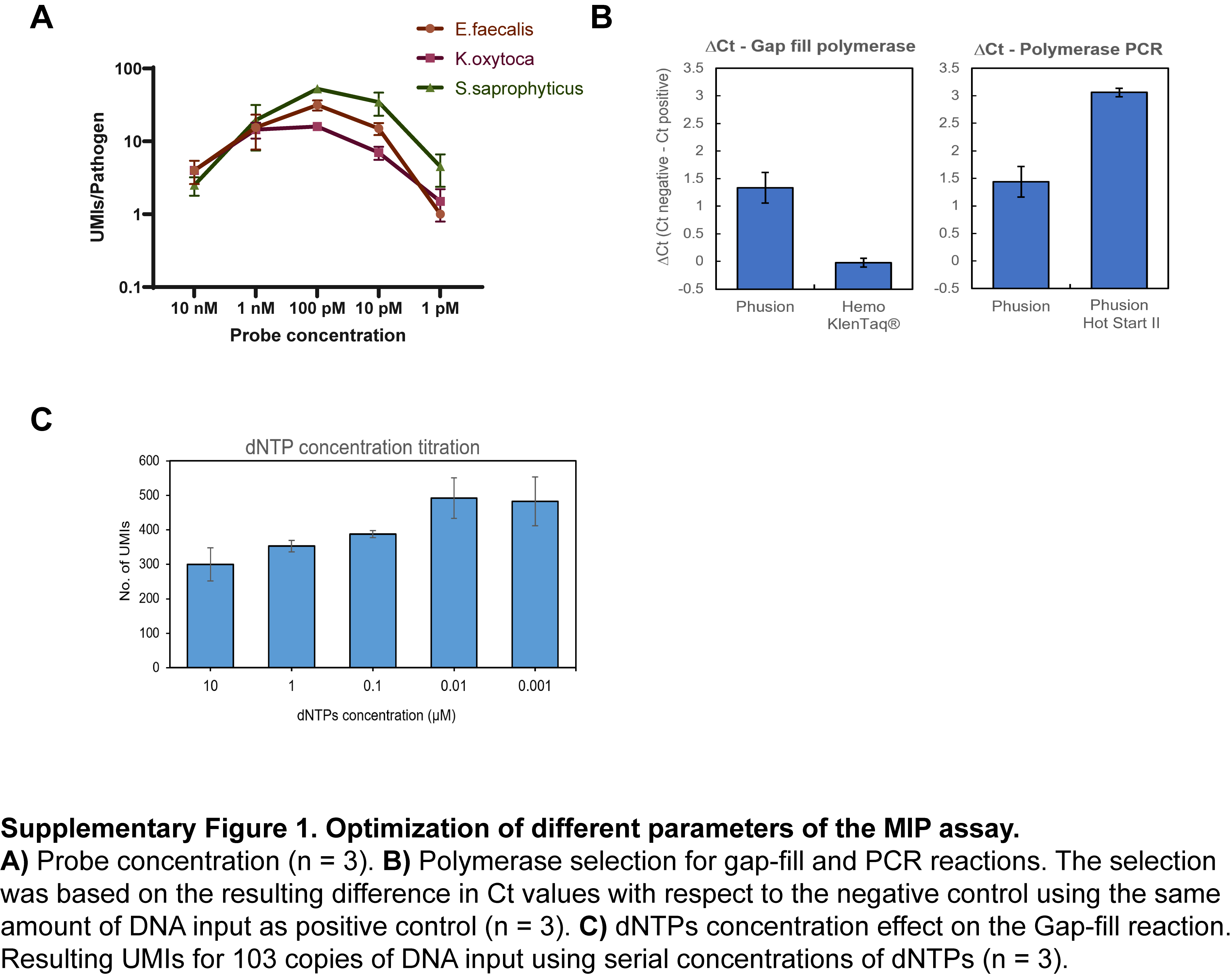

Supplement: Supplementary file 6 — Supplementary Material 6 [file 12896_2023_804_MOESM6_ESM.tif]

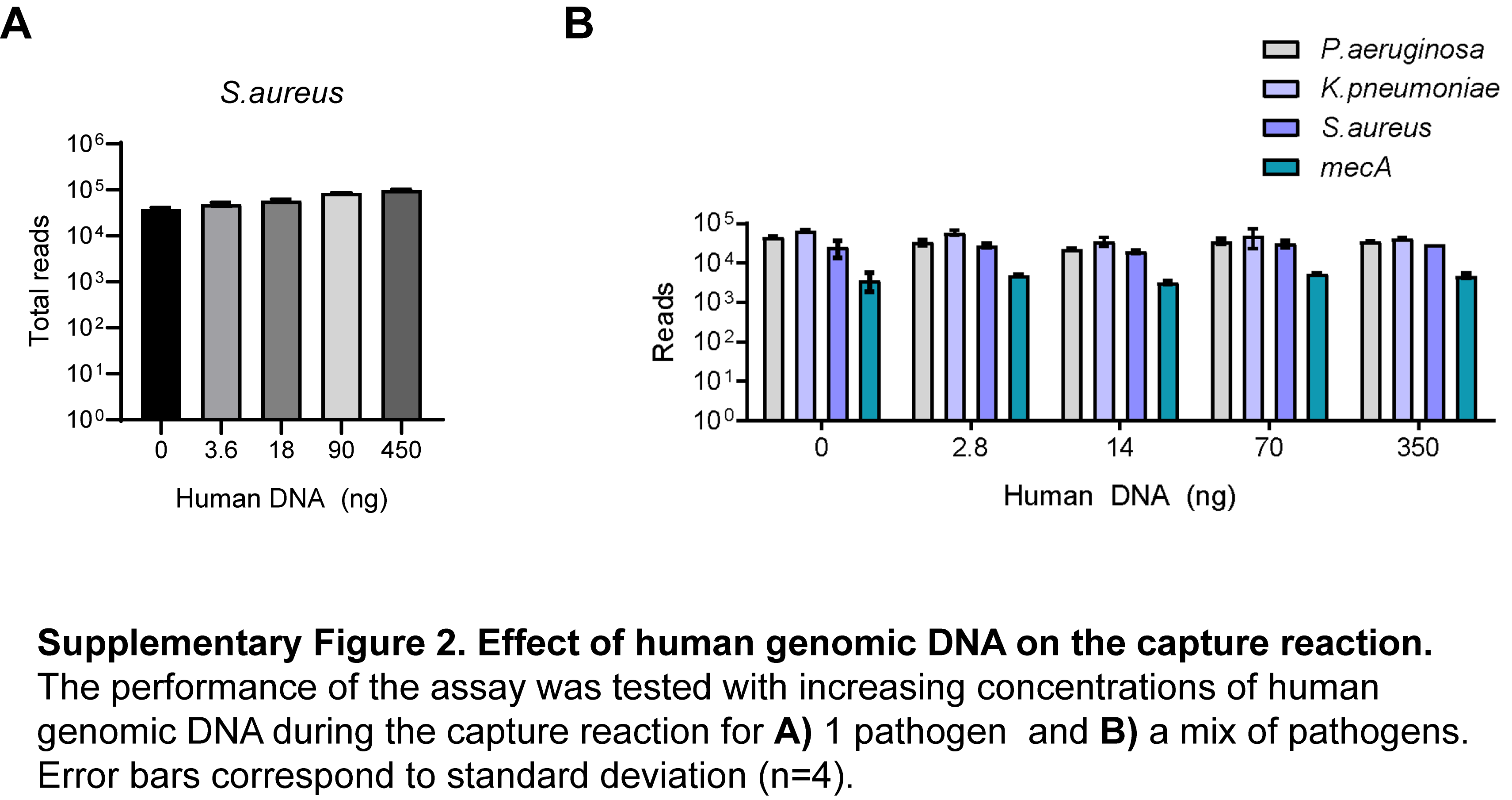

Supplement: Supplementary file 7 — Supplementary Material 7 [file 12896_2023_804_MOESM7_ESM.tif]

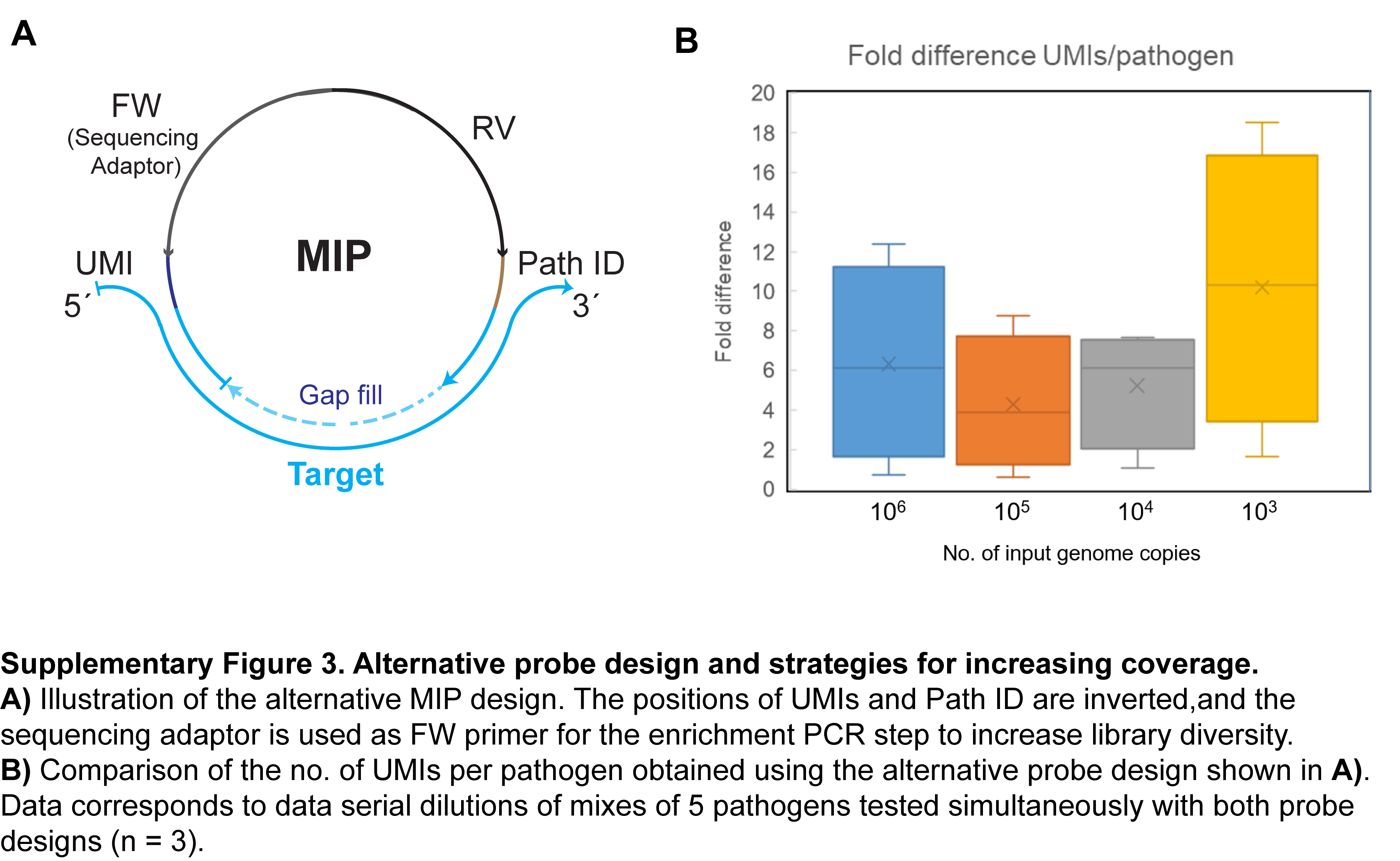

Supplement: Supplementary file 8 — Supplementary Material 8 [file 12896_2023_804_MOESM8_ESM.tif]

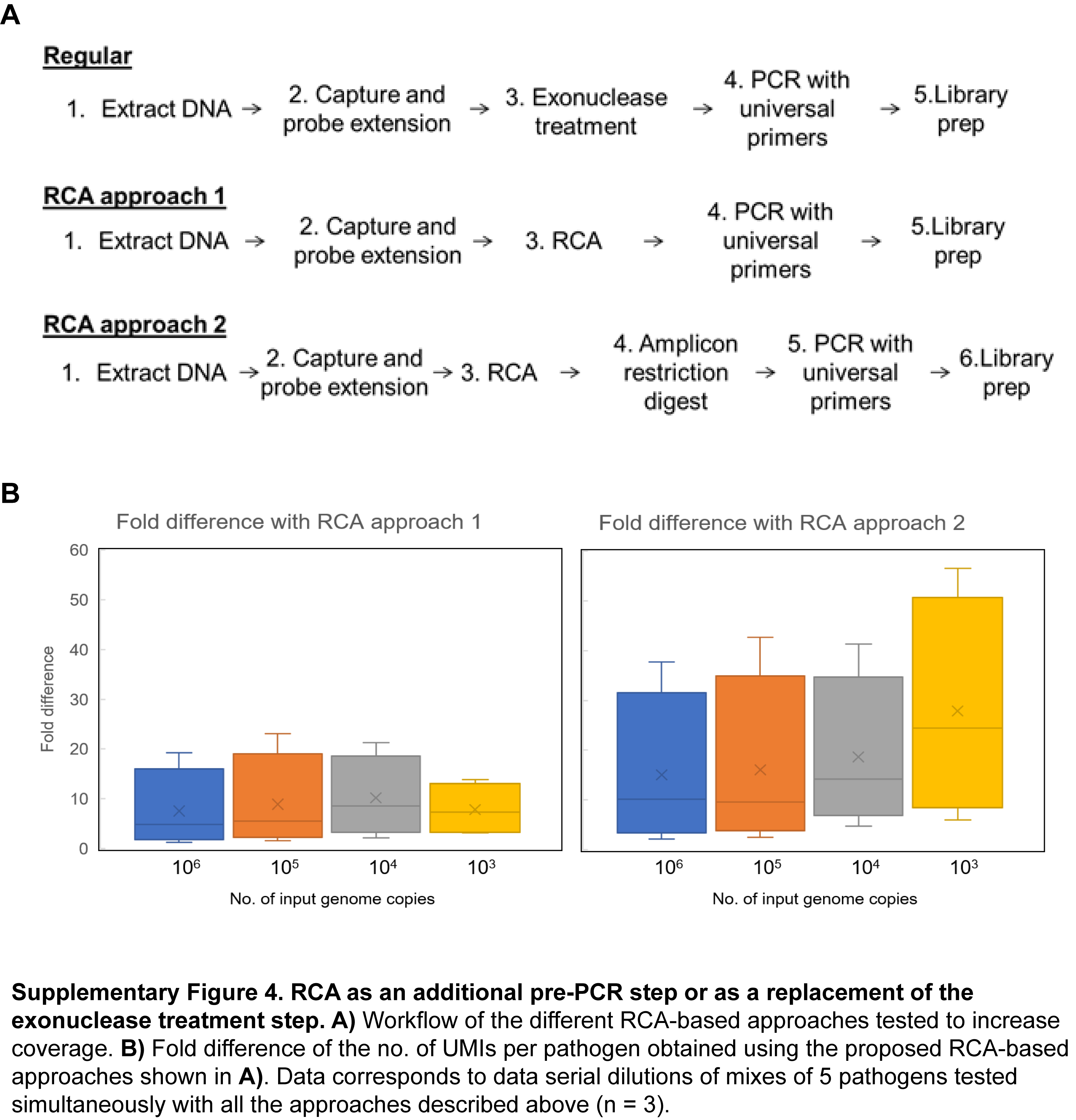

Supplement: Supplementary file 9 — Supplementary Material 9 [file 12896_2023_804_MOESM9_ESM.tif]
